# Reversible differentiation of immortalized human bladder smooth muscle cells accompanied by actin bundle reorganization

**DOI:** 10.1371/journal.pone.0186584

**Published:** 2017-10-19

**Authors:** Naohiro Hashimoto, Tohru Kiyono, Fumihito Saitow, Minoru Asada, Masaki Yoshida

**Affiliations:** 1 Department of Regenerative Medicine, Research Institute, National Center for Geriatrics and Gerontology, Oobu, Aichi, Japan; 2 Division of Carcinogenesis and Cancer Prevention, National Cancer Center Research Institute, Chuo-ku, Tokyo, Japan; 3 Department of Pharmacology, Nippon Medical School, Bunkyo-ku, Tokyo, Japan; 4 Department of Urology, Hospital, National Center for Geriatrics and Gerontology, Oobu, Aichi, Japan; University of Minnesota Medical Center, UNITED STATES

## Abstract

Previous studies have shown that phenotypic modulation of smooth muscle cells (SMCs) plays a pivotal role in human diseases. However, the molecular mechanisms underlying the reversible differentiation of SMCs remain elusive particularly because cultured SMCs that reproducibly exhibit bidirectional phenotypic modulation have not been established. Here we established an immortalized human bladder SMC line designated as hBS11. Under differentiation-inducing conditions, hBS11 cells underwent smooth muscle differentiation accompanied by the robust expression of smooth muscle differentiation markers and isoform-dependent reorganization of actin bundles. The cholinergic receptor agonist carbachol increased intracellular calcium in differentiated hBS11 cells in an acetylcholine muscarinic receptor-dependent manner. Differentiated hBS11 cells displayed contractile properties depending on the elevation in the levels of intracellular calcium. Depolarization of membrane potential triggered inward sodium current in differentiated hBS11 cells. However, differentiated hBS11 cells lost the differentiated phenotype and resumed mitosis when re-fed with growth medium. Our study provides direct evidence pertaining to the human bladder SMCs being able to retain the capacity of reversible differentiation and that the reorganization of actin bundles is involved in the reinstatement of contractility. Moreover, we have established a human SMC line retaining high proliferating potential without compromising differentiation potential.

## Introduction

Many previous studies on the regeneration of injured arteries *in vivo* and primary cultured smooth muscle cells (SMCs) *in vitro* showed that fully differentiated SMCs phenotypically switch to a proliferating state [1–3]. Extensive evidence indicates that alterations in the normal function and phenotypic modulation of SMCs play a key role in numerous diseases and during the injury repair of smooth muscle tissues, including blood vessel, trachea, stomach, small intestine, urinary bladder, and uterus [4]. Understanding of the mechanisms that regulate SMC differentiation will aid in the study of the etiology and therapeutic interventions of human diseases. However, the cellular events and molecular mechanisms related to the phenotypic modulation of SMCs remain elusive [1–3].

SMC cultures are an excellent tool for the study of cellular events and molecular mechanisms by which the phenotypic plasticity is controlled; many SMC systems have been previously analyzed. However, SMC systems that reproducibly exhibit bidirectional phenotypic modulation between undifferentiated and differentiated states have not been developed [5]. During the early period of primary culture, differentiated SMCs spontaneously change their phenotype to undifferentiated ones [1, 2]. However, the factors or stimuli that trigger this modulation, i.e., retrograde differentiation, are unknown because freshly prepared differentiated SMCs immediately undergo dedifferentiation with irreversible loss of the smooth muscle differentiation potential [1, 2]. These SMCs that are unable to differentiate are called dedifferentiated cells [1–3]. The characteristics of the dedifferentiated cells were previously investigated [6–8]. In contrast to the dedifferentiated cells, undifferentiated and proliferating SMCs that retain their differentiation potential have not been characterized. Previous studies were unable to maintain the proliferation of SMCs without compromising their differentiation potential. The molecular mechanisms underlying smooth muscle differentiation have not been explored because of the lack of proper SMC cultures.

To develop reproducible *in vitro* cellular models, cell lines have been established from human [9, 10] and animal SMCs [11, 12]; however, these cells lines do not show bidirectional phenotypic modulation. The establishment of *in vitro* human SMC systems is required for the causal analysis of symptoms and therapeutic approaches of human smooth muscle diseases, including bladder dysfunction and atherosclerosis. In addition to dedifferentiation, human SMCs exhibit a limited proliferation capacity, which is likely due to the activation of the p16^INK4a^-the retinoblastoma protein (Rb) pathway under *in vitro* culture conditions [13–15]. Previous studies reported that human SMC lines have inadvertently acquired the capacity for replication *in vitro*. These cell lines may have undergone multiple alterations outside of the p16^INK4a^-Rb pathway. The previously established human SMC lines exhibit an abortive karyotype and/or extraordinarily prolonged doubling time [10, 16].

Direct blockage of the p16^INK4a^-Rb pathway is a probable way to establish SMC lines without compromising normal karyotype and differentiation potential. The establishment of multiple types of immortalized SMC line derived from different smooth muscle tissues will enable us to explore distinct characteristics and functions of vascular and visceral SMCs. Functional changes in bladder SMCs, i.e., bladder detrusor cells, can impair urinary bladder function and result in lower urinary tract symptoms and bladder outlet obstruction [17]. However, the cellular and molecular mechanisms that control the unique functions of bladder SMCs have yet to be identified.

In the present study, we immortalized primary cultured human bladder SMCs by expressed mutated CDK4, cyclinD1, and human telomerase (hTERT) genes to establish a reproducible SMC system to study the regulatory mechanisms of differentiation. Our three-factor method [15] directly inactivates Rb and prevents telomere shortening, resulting in the immortalization of bladder SMCs without compromising cellular functions. The newly established human bladder SMC line hBS11 retained the potential to reversibly shuttle between undifferentiated and differentiated states. We also optimized the culture conditions for defined SMC phenotypes. Notably, the isoform-dependent reorganization of actin cytoskeletal bundles plays an important role in smooth muscle differentiation.

## Materials and methods

### Cell culture

Primary cultured and immortalized human bladder smooth muscle cells were maintained in standard culture vessels at 37°C under 10% CO_2_ in Smooth Muscle Cell Growth Medium 2 (SMC-GM2, c-22062; PromoCell, Heidelberg, Germany) or primary cultured myocyte growth medium (pmGM) [18], which comprised Dulbecco’s modified Eagle’s medium–high glucose (hDMEM, D6429; Sigma, St. Louis, MO) supplemented with 20% fetal bovine serum (FBS) and 2% Ultroser G (Biosepra, Cedex-Saint-Christophe, France). For the induction of smooth muscle differentiation, cells were plated at a density of approximately 10 cells/mm^2^ in hDMEM supplemented with 20% FBS. The next day, cells were rinsed with phosphate buffered saline (PBS) and the medium was changed to primary cultured myocyte differentiation medium (pmDM) [18], which comprised the chemically defined medium TIS [19, 20] supplemented with 2% FBS.

### Immortalization of human smooth muscle cells

Primary cultured human bladder smooth muscle cells (HBdSMCs) derived from a 68-year-old white man were purchased from PromoCell (Lot 6110301.10) and transduced with recombinant lentiviruses and encoding human cyclin D1, human mutant CDK4, and human telomerase [15]. The backbone lentivirus vector, CSII-CMV-RfA, was a kind gift from Hiroyuki Miyoshi (Keio University, Yokohama, Japan). The immortalized human SMC line hBS11 will be available from RIKEN BioResorce Center (http://www.brc.riken.go.jp) within a few months after publication.

### Cell proliferation assay

Approximately 1,000 hBS11 cells were plated in each well of a 96-well plate containing hDMEM supplemented with 10% FBS. The cells attached to the culture vessels within 24 h after plating. The next day, the medium was changed to a test medium and cultured for another 3 days. Cells were fixed in 4% paraformaldehyde (PFA), and nuclei were stained with 2,4-diamidino-2-phenylindole dihydrochloride *n*-hydrate (DAPI) (1 μg/ml, Sigma). Then number of nuclei was quantified using an In Cell Analyzer 2000 (GE Healthcare, Piscataway, NJ).

### Immunoblotting analysis

The sample preparation and immunoblot analysis was performed as previously described [19, 20]. Immune complexes were detected by colorimetry with SIGMAFAST BCIP/NBT tablets (Sigma). Primary antibodies included mouse monoclonal antibodies for calponin (hCP; Sigma), high molecular weight caldesmon, i.e., *h*-caldesmon (h-CD, M3557; DAKO, Carpinteria, CA), α-smooth muscle actin [21] (1A4, undiluted culture supernatant), γ-smooth muscle actin [22] (20D2, undiluted culture supernatant), and β-cytoplasmic actin [23] (4C2, undiluted culture supernatant); rabbit monoclonal antibodies for muscarinic acetylcholine receptor M2 (Abcam, Cambridge, England), muscarinic acetylcholine receptor M3 (Abcam), and β-actin (13E5; Cell Signaling Technology, Beverly, MA); and rabbit polyclonal antibodies for smooth muscle myosin heavy chain 11 (ab53219; Abcam) and β-tubulin (Cell Signaling Technology). Secondary antibodies included alkaline phosphatase-labeled antibodies to mouse or rabbit immunoglobulin G (DAKO). Immune complexes on PVDF membranes (Fluoro Trans W; Pall, Port Washington, NY) were scanned with a digital scanner (GT-9700F; Epson, Suwa, Japan) and then post-processed using Adobe Photoshop (Adobe Systems, San Jose, CA). The actin isoform specific antibodies were kind gifts from Christine Chaponnier (University of Geneva, Geneva, Switzerland).

### Immunofluorescence analyses

Cells were grown in culture dishes, then fixed, permeabilized, and processed for immunostaining as previously described [24, 25]. Primary antibodies included mouse monoclonal antibodies for α-smooth muscle actin (1A4, undiluted culture supernatant; A5228, Sigma), γ-smooth muscle actin (20D2, undiluted culture supernatant), and β-cytoplasmic actin (4C2, undiluted culture supernatant), and a rabbit polyclonal antibody for connexin 43 (Sigma). Secondary antibodies included Cy3-labeled donkey antibodies to mouse or rabbit immunoglobulin G (Jackson ImmunoResearch Laboratory, Bar Harbor, ME), Alexa Fluor 488-labeled donkey antibodies to mouse or rabbit immunoglobulin G (Jackson ImmunoResearch Laboratory), Alexa Fluor 488-labeled goat antibodies to mouse immunoglobulin G1 (Jackson ImmunoResearch Laboratory), and Cy3-labeled goat antibodies to mouse immunoglobulin G2a (Jackson ImmunoResearch Laboratory). Cell nuclei were stained with DAPI (1 μg/ml). Actin filaments were stained with Alexa Fluor 546-labeled phalloidin (Life Technologies) as previously described [26]. Samples were visualized using an inverted microscope (model IX71; Olympus, Tokyo, Japan) and a CCD camera (DP70; Olympus). Images were post-processed using Adobe Photoshop (Adobe Systems, San Jose, CA).

### Time-lapse recording

Cells were cultured in a humidified chamber (Tokai Hit, Fujinomiya, Japan) maintained at 37°C under 10% CO_2_. Time-lapse images were taken using an inverted microscope (BZ9000; Keyence, Osaka, Japan) with a 20x Plan Apo Fluor objective lens (Nikon, Tokyo, Japan) [27, 28].

### Live imaging of intracellular calcium

Cells were preloaded with the calcium sensitive dye Fluo-4 AM (Dojindo, Kumamoto, Japan) according to the manufacturer’s instructions provided with the Calcium Kit-Fluo 4 (CS22; Dojindo). Cells were incubated with 2.5 μM Fluo-4 AM in Recording Buffer for 1 h. The medium was switched to an isotonic Krebs-Ringer solution [10 mM D-glucose, 0.5 mM magnesium chloride, 4.56 mM potassium chloride, 119.78 mM sodium chloride, 0.70 mM sodium phosphate dibasic, 1.50 mM sodium phosphate monobasic, 2.8 mM and calcium chloride] or a high potassium-containing/calcium-depleted Krebs–Ringer solution [HiK/Ca(-)-Krebs-Ringer; 10 mM D-glucose, 0.5 mM magnesium chloride, 90 mM potassium chloride, 34.34 mM sodium chloride, 0.70 mM sodium phosphate dibasic, and 1.50 mM sodium phosphate monobasic]. Next, carbachol (final concentration of 1 mM) or calcium chloride (final concentration of 2.8 mM) was added to the isotonic or HiK/Ca(-)-Krebs-Ringer solution, respectively. Time-lapse images were obtained with epifluorescence microscopy using FITC settings [27].

For the quantification of intracellular calcium, cells were labeled with Fluo-4 AM, washed with isotonic Krebs-Ringer solution, and then carbachol was puff applied for 30 s onto the cells through a glass pipette controlled by a picospritzer (PV830, World Precision Instruments, Sarasota, FL). Digital fluorescence imaging was performed using a 2-photon confocal microscope system (FV1000; Olympus) essentially as previously described [29]. Fluorescence was excited using an 800 nm wavelength pulse laser and emissions were detected using a barrier filter (495–540 nm). Fluorescence time courses were then recorded in the frame scan mode (320 × 320 pixels), and images were sampled at 1 s intervals. Intracellular calcium concentration was estimated as percent changes in the fluorescence intensity over resting levels (ΔF/F_0_ × 100). Data were analyzed using the software ImageJ (National Institutes of Health, Bethesda, MD) and KyPlot (KyensLab Inc., Tokyo, Japan).

### Electrophysiology

hBS11 cells were cultured on a glass coverslip for 14–19 days in pmDM. Voltage-dependent sodium currents were recorded using the whole-cell patch-clamp mode. Coverslip was set on a submersion-type recording chamber perfused with isotonic Krebs-Ringer solution bubbled with O_2_ gas. Borosilicate glass-patch electrodes (World Precision Instruments, Sarasota, FL) with a resistance of 4–6 MΩ when filled with an internal solution containing 150 mM cesium methanesulphonate, 5 mM potassium chloride, 0.5 mM ethylene glycol tetra acetic acid, 10 mM 4-(2-hydroxyethyl)-1-piperazineethanesulfonic acid, 5 mM Mg-ATP, and 0.4 mM Na-GTP (pH 7.4) were used. Membrane currents in the whole-cell configuration were acquired using Axon 700B Multiclamp amplifier and pClamp acquisition software (Molecular Devices, Sunnyvale, CA). The peak sodium current was recorded during a series of depolarizing voltages at holding potentials from −80 mV to 80 mV (200-ms pulse duration) in 10 mV increments at 6 s intervals. Leak currents were subtracted using the online P/8 protocol. The whole-cell current voltage (I-V) curves were obtained by measurement of the peak inward current at each depolarizing potential. To examine whether the sodium current was blocked by tetrodotoxin (TTX, Wako, Tokyo, Japan), TTX-containing extracellular solution was delivered by bath application during the recording of depolarization-induced sodium currents (stepped from −80 to 0 mV). Data were analyzed using the software Clampfit (Molecular Devices) and KyPlot.

### Live imaging of F-actin

F-actin in live cells was labeled with 100 or 200 μM of SiR-A for 2 h according to the manufacturer’s instructions (SiR-actin kit, Cytoskeleton, Denver, CO). SiR-A is s jasplakinolide conjugated with the fluorophore silicon rhodamine. The labeled hBS11 cells were cultured in SiR-actin-free pmDM for 1–3 days to avoid possible acute effects of SiR-A on actin dynamics because jasplakinolide inhibits actin depolymerization. The medium was switched to isotonic Krebs-Ringer solution or Hi-K/Ca(-)-Krebs-Ringer solution, and time-lapse images were obtained using phase contrast and/or epifluorescence microscopy BZ9000 with Cy5 settings.

### Quantification of globular and filamentous actin

Globular (G-) and filamentous (F-) actin were separated from cell lysates using the G-actin / F-actin In Vivo Assay Kit (Cytoskeleton, Denver, CO) according to the manufacturer’s instructions. Fractions of G-actin and F-actin derived from 10 μg of total lysates were processed for immunoblotting with actin isoform-specific antibodies. Immune complexes on the membranes were scanned by a digital scanner and then quantified using Image J software.

### Karyotype analysis

After incubation in pmGM supplemented with 0.5 μM colcemid at 37°C for 3 h, cells were trypsinized and incubated in 0.5 ml of 1% sodium citrate for 15 min. This was followed by the addition of 0.5 ml of Carnoy’s fixative (methanol: acetic acid, 3:1 by vol). The fixed cells were centrifuged and resuspended in 0.5 ml of Carnoy’s fixative. Metaphase chromosomes were stained with a 10% Giemsa solution (Wako Pure Chem., Osaka, Japan) for 10 min. Samples were visualized using an inverted microscope (model IX71; Olympus) and a CCD camera (DP70; Olympus). Images were post-processed using Adobe Photoshop.

### EdU labeling and detection

DNA synthesizing cells were detected with a Click-iT EdU Alexa Fluor 488 Imaging Kit (Thermo Fisher Scientific, Waltham, MA). Cells were incubated with 10 μM 5-ethynyl-2'-deoxyuridine (EdU) for the last 4 h of each culture, fixed in 4% paraformaldehyde for 10 min, and then subjected to the Click-It reaction according to the manufacturer’s instructions. Cell nuclei were stained with DAPI. Samples were visualized, and the DAPI- and EdU-positive nuclei were quantified using an In Cell Analyzer 2000. EdU-positive nuclei were detected in three independent wells of a 24-well plate for each treatment.

### Expression microarrays

The total RNA of hBS11 cells was extracted and purified using Qiagen RNeasy minicolumns (Qiagen, Venlo, Netherlands). Agilent Cy3-CTP labelled cRNA was produced from 100 ng of total RNA using the Agilent Low Input Quick Amp Labeling Kit (Agilent, Santa Clara, CA). After labeling and cRNA purification, cRNA was quantified using the NanoDrop ND-1000 UV-VIS Spectrophotometer (Thermo Fisher Scientific). Cy3- labeled cRNA (600 ng) was hybridized per individual 60K array on the catalogue Agilent SurePrint G3 Human GE Microarray 8x60K ver. 3.0 (G4851C) for 17 h at 65°C (10 rpm). After hybridization, arrays were washed consecutively with Agilent gene expression wash buffer one for 1 min at room temperature, and Wash buffer two for 1 min at 37°C. Slides were scanned immediately after washing using an Agilent Scanner and Scan Control software ver. A.8.5.1. The scan resolution was 3 μm, and a 20-bit dynamic range TIFF file was used to avoid saturated features. Scan data were extracted with Feature Extraction v. 10.7.3.1 software using the GE1_107_Sep09 protocol. Extracted signal intensities were analyzed using GeneSpring 13.1.1 software (Agilent), and the datasets were normalized using a standard percentile shift procedure, which accounts for interchip variability by adjusting the intensity distribution to the 75th percentile. Microarray analysis was performed twice and representative data were shown.

### Ethics statement

Institutional Review board approval was not needed for the present study because non-immortalized human bladder smooth muscle cells were purchased from the supplier PromoCell. All the experimental procedures in the present study were carried out in accordance with the guidelines approved by the ethics committee of National Center for Geriatrics and Gerontology.

## Results

### Cell cycle drivers and telomerase immortalize primary cultured human bladder smooth muscle cells

**Fig 1:**
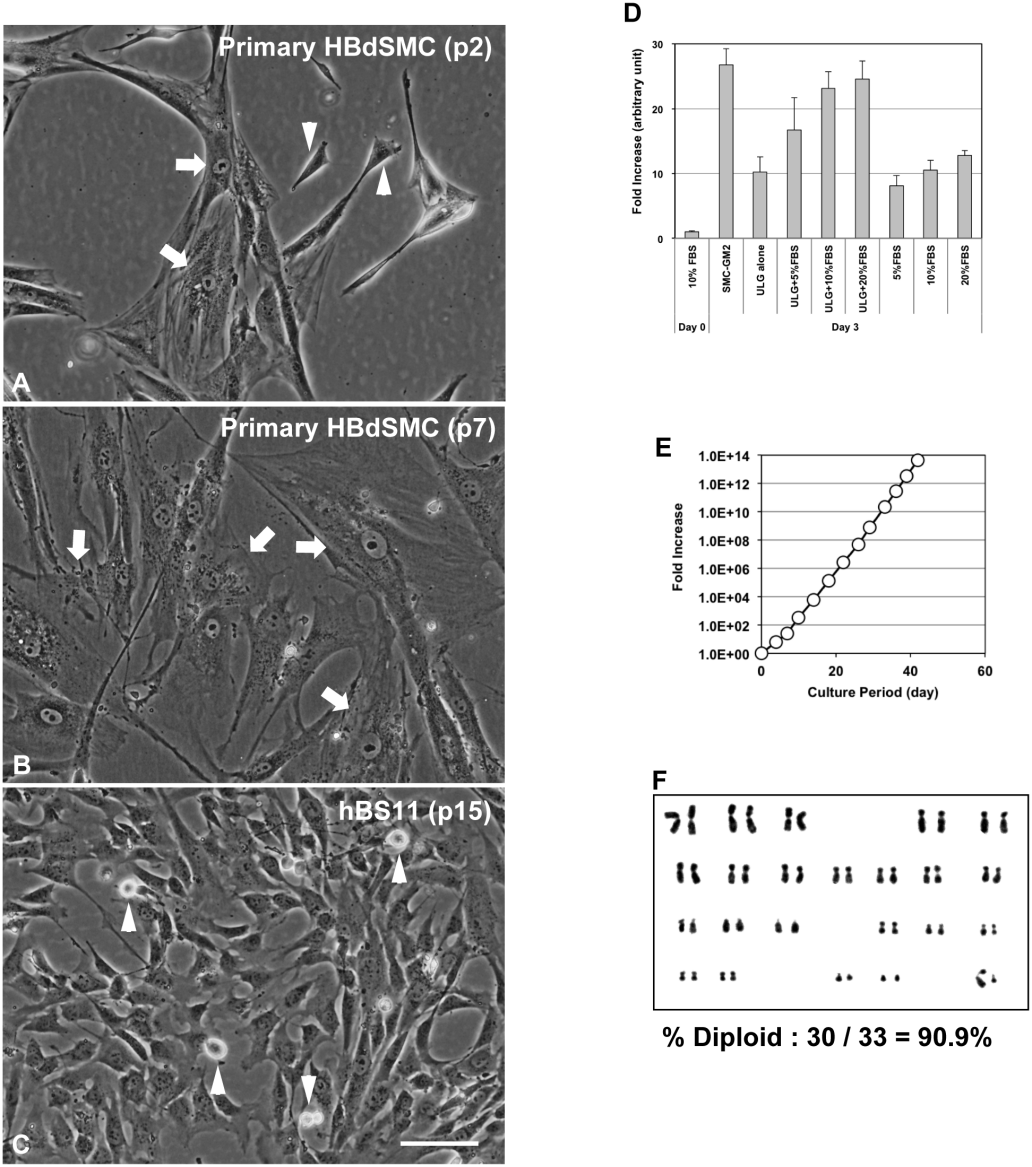
Growth properties of immortalized human bladder smooth muscle cells. (A-C) Morphological features of human bladder smooth muscle cells. Parental primary cultured human bladder smooth muscle cells at passage 2 (A) in Smooth Muscle Cell Growth Medium 2 contained heterogeneous cell populations that included compact cells (arrow heads) and spreading cells (arrows). Parental primary cultured human bladder smooth muscle cells at passage 7 (B) contained extensively spreading cells (arrows) and a loss of proliferative potential even when cultured in pmGM. Immortalized human bladder smooth muscle hBS11cells (C) exhibited a compact and rhomboid shape. Arrow heads represent mitotic cells. The images in (A-C) are shown at the same magnification. Scale bar, 50 μm. (D) Optimization of culture medium components for the growth of immortalized human bladder smooth muscle cells. hBS11 cells were plated in Dulbecco’s modified Eagle’s medium–high glucose (hDMEM) supplemented with 10% FBS. The next day (day 0), the medium was switched to a test medium, and cells were cultured for another 3 days. The number of nuclei was counted and normalized to the day 0 values. Averages and standard deviations were estimated from four independent cultures for each treatment. hDMEM was used as a basal medium except for SMC-GM2. FBS, fetal bovine serum; ULG, 2% Ultroser G. (E) The lifespan of immortalized human bladder smooth muscle cells. The number of multiclonal cells (hBS11) between passage 7 and 19 was calculated. Day 0 of the culture period represents when the cells were plated for passage 7. The fold increase was estimated by normalizing to the cell number at day 0. (F) Karyotype analysis of immortalized human bladder smooth muscle cells. hBS11 cells (passages 11 and 12) were treated with colcemid (0.5 μM) for 3 h. Metaphase chromosomes 46, XY were visualized using Giemsa staining.

The proliferation capacity of primary cultured human bladder SMCs (HBdSMCs) severely declined during early passages (approximately passage 7). HBdSMC cultures at passage 2 contained a heterogeneous cell population that included proliferating compact cells and extensively spreading cells (Fig 1A). Time-lapse recordings showed that compact cells in the HBdSMC cultures rapidly divided with cell cycle interval of 17.2 ± 5.2 h (n = 22), whereas the spreading cells divided once or not at all (S1 Video). The postmitotic compact cells lost proliferative capacity, underwent hypertrophy, and then gave rise to the spreading cells (S1 Fig). Thus, by passage 7, the parental HBdSMC cultures contained only non-proliferating spreading cells (Fig 1B). The supplier reported a population doubling time of the parental HBdSMCs as 38.7 h. This value likely reflects both dividing and non-dividing cells.

A previous study suggested that activation of the p16^INK4a^-Rb pathway triggers precocious growth arrest in human skeletal muscle progenitor cells that is independent from telomere shortening [15]. To immortalize HBdSMCs, cells were infected with recombinant lentiviruses encoding hTERT, mutated CDK4, and cyclin D1 as previously described [15]. Then, the infected cells continued to proliferate even after passage 7. The virtually immortalized HBdSMCs, designated as hBS11, exhibited a homogeneous population containing exclusively proliferating compact cells (Fig 1C). The cell cycle interval, directly calculated from time-lapse recordings, was 18.0 ± 3.2 h (n = 39) (S2 Video). Thus, hBS11 cells retain the rapidly dividing capacity of the proliferating subpopulation of parental cells.

Initially, primary cultured HBdSMCs and hBS11 cells were cultured in Smooth Muscle Cell Growth Medium 2 (SMC-GM2) supplemented with 5% fetal bovine serum (FBS), vanadate, epidermal growth factor, basic fibroblast growth factor, and insulin according to the manufacturer’s instructions. Both the parental HBdSMCs and hBS11 cells required a significant amount of time before they attached to the substratum of the culture vessels. Some floating cells remained even 24 h after seeding. To optimize culture conditions for cell attachment and exponential growth, the growth supporting capacity of culture media containing a high concentration of FBS was compared with SMC-GM2. High concentrations of FBS (>10%) enhanced cell attachment. Once cells attached to culture vessels, their proliferation was highly dependent on supplementation with FBS and Ultroser G serum substitute containing steroid and growth factors (Fig 1D). Therefore, we used primary myocyte growth medium (pmGM) [18], which comprises Dulbecco’s modified Eagle’s medium–high glucose (hDMEM) supplemented with 20% FBS and 2% Ultroser G, in subsequent experiments.

hBS11 cells exhibited continuous cell proliferation for more than 50 population doublings under optimized culture conditions *in vitro* (Fig 1E). A chromosome analysis of hBS11 cells revealed a normal 46XY diploid karyotype (Fig 1F). Thus, hBS11 cells inherit the rapidly dividing capacity and normal karyotype from the proliferating subpopulation of parental HBdSMCs.

### Immortalized bladder smooth muscle cells retain their differentiation potential

**Fig 2:**
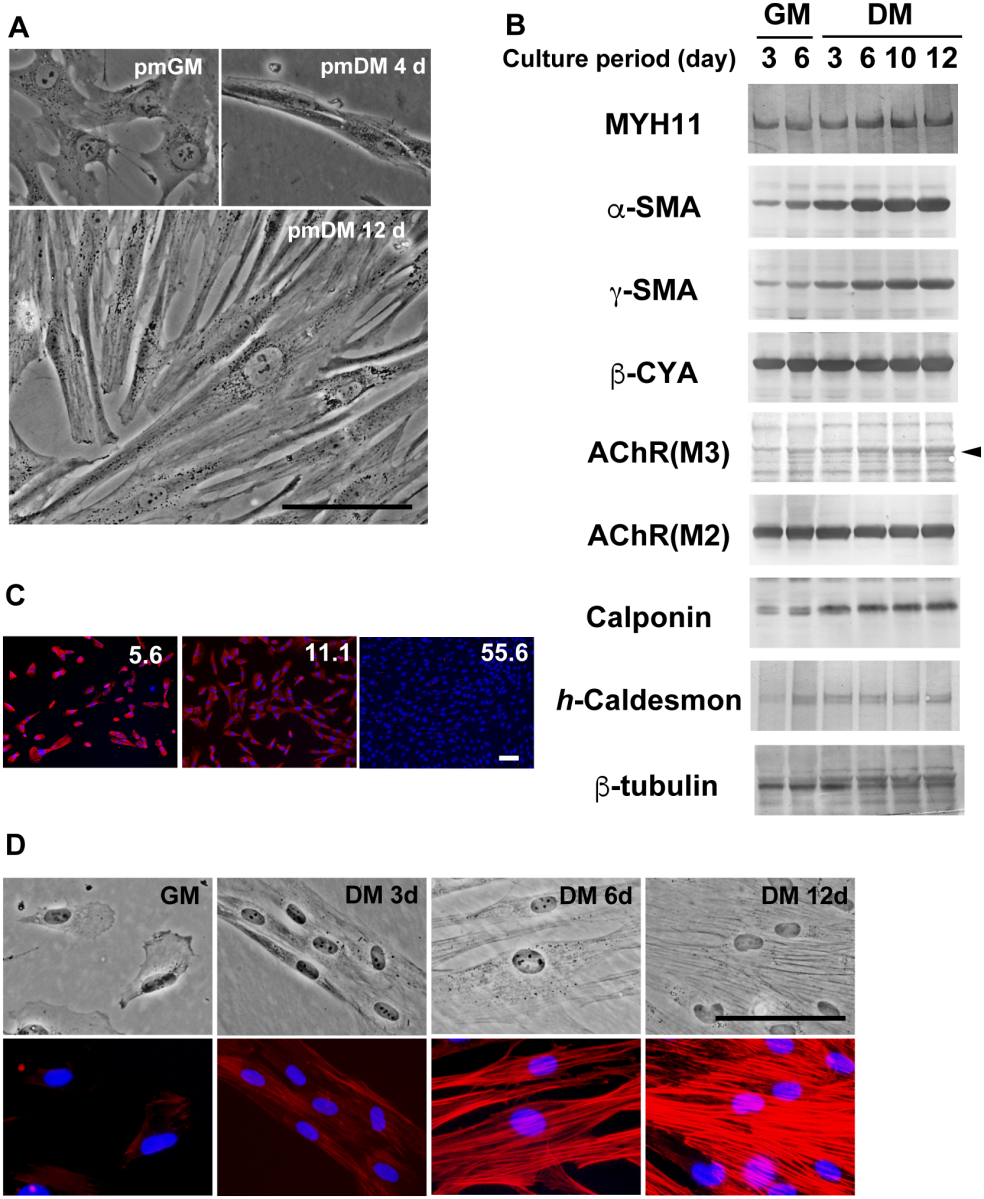
Smooth muscle differentiation of immortalized human bladder smooth muscle cells. (A) hBS11 cells were cultured in pmGM for 3 days or pmDM for 4 and 12 days. Phase contrast images were taken at the same magnification. Scale bar, 100 μm. (B) hBS11 cells were cultured in pmGM for 3 and 6 days or pmDM for 3, 6, 10, and 12 days. Ten micrograms of total protein was subjected to immunoblotting analysis with antibodies for myosin heavy chain 11 (MYH11), α-smooth muscle actin (α-SMA), γ-smooth muscle actin (γ-SMA), β-cytoplasmic actin (β-CYA), acetylcholine muscarinic receptor 3 (Ach(M3)), acetylcholine muscarinic receptor 2 (Ach(M2)), calponin, *h*-caldesmon, and β-tubulin. (C) hBS11 cells were plated at a density of 5.6, 11.1, or 55.6 cells/mm^2^ in hDMEM supplemented with 20% FBS. The cells were then cultured in pmDM for 6 days. The cells were subjected to immunofluorescence analysis with antibodies for α-SMA (red). Nuclei were stained with DAPI (blue). Scale bar, 100 μm. (D) hBS11 cells were cultured in pmGM for 3 days or pmDM for 3, 6, and 12 days. Phase contrast images (upper row) and fluorescent images with Alexa 546-conjugated phalloidin (red in lower row) of the same fields are shown in each column. Nuclei were stained with DAPI (blue in lower row). Scale bar, 100 μm.

The morphological features of hBS11 cells depended on the culture conditions (Fig 2A). hBS11 cells had a compact and rhomboid shape in pmGM. In contrast, the cells adopted a spindle-like shape when cultured for three or four days in pmDM, a myocyte differentiation medium [18]. The cells underwent hypertrophy after prolonged culturing in pmDM. Cell proliferation and migration was severely attenuated in pmDM, and stable cell-to-cell contact was subsequently established. In addition, a high cell density disrupted the morphological changes during culturing.

Our data suggest that hBS11 cells undergo smooth muscle differentiation when cultured at a low cell density in pmDM. Exponentially growing hBS11 cells expressed smooth muscle differentiation marker proteins, including myosin heavy chain 11 (MYH11), α-smooth muscle actin (α-SMA), γ-smooth muscle actin (γ-SMA), and calponin at relatively lower levels (lane 1 in Fig 2B). Then the expression of α-SMA, γ-SMA, and calponin was markedly increased and the smooth muscle differentiation–specific isoform of caldesmon, *h*-caldesmon, appeared in cells cultured in pmDM (lanes 3–6 in Fig 2B). The expression of acetylcholine muscarinic receptor 3, AChR(M3), was low but increased when cultured in pmDM. In contrast, β-cytoplasmic actin (β-CYA) and acetylcholine muscarinic receptor 2, AChR(M2), were robustly expressed independently from culture conditions. Postconfluent cultures grown in pmGM contained a few cells that showed the smooth muscle differentiation phenotype (lane 2 in Fig 2B, S2 Fig).

To determine the effects of cell density on smooth muscle differentiation, hBS11 cells were plated at a high or low density and cultured in pmDM for 6 days. Increased α-SMA expression and the development of an elongated shape were cell density-dependent (Fig 2C). Thus, low cell density cultures may facilitate hypertrophy by reserving space for cell spreading. Indeed, hypertrophy is required for complete SMC differentiation. The combination of low cell density and serum deprivation was essential for optimized smooth muscle differentiation-inducing conditions. Notably, hBS11 cells retain the ability to undergo smooth muscle differentiation under the appropriate culture conditions.

### Actin bundles are reorganized during smooth muscle differentiation

The dynamics of filamentous actins (F-actins) in hBS11 cells during smooth muscle differentiation was studied using Alexa Fluor 546-conjugated phalloidin. A few short actin bundles (fibers) were observed in lamellipodia and filopodia and beneath the plasma membrane of proliferating hBS11 cells (GM in Fig 2D). Actin bundles began to develop in spindle-shaped hBS11 cells within 3 days of culturing in differentiation medium (DM 3 d in Fig 2D). Next, hBS11 cells spread and underwent hypertrophy (DM 6 d and 12 d in Fig 2D). The number and length of actin bundles simultaneously increased as hBS11 cells became hypertrophic. In addition, the signal intensity of phalloidin-stained actin bundles was markedly increased during differentiation, suggesting that F-actin increases during smooth muscle differentiation.

### The recruitment of α-SMA contributes to actin bundle reorganization during the later stages of smooth muscle differentiation

**Fig 3:**
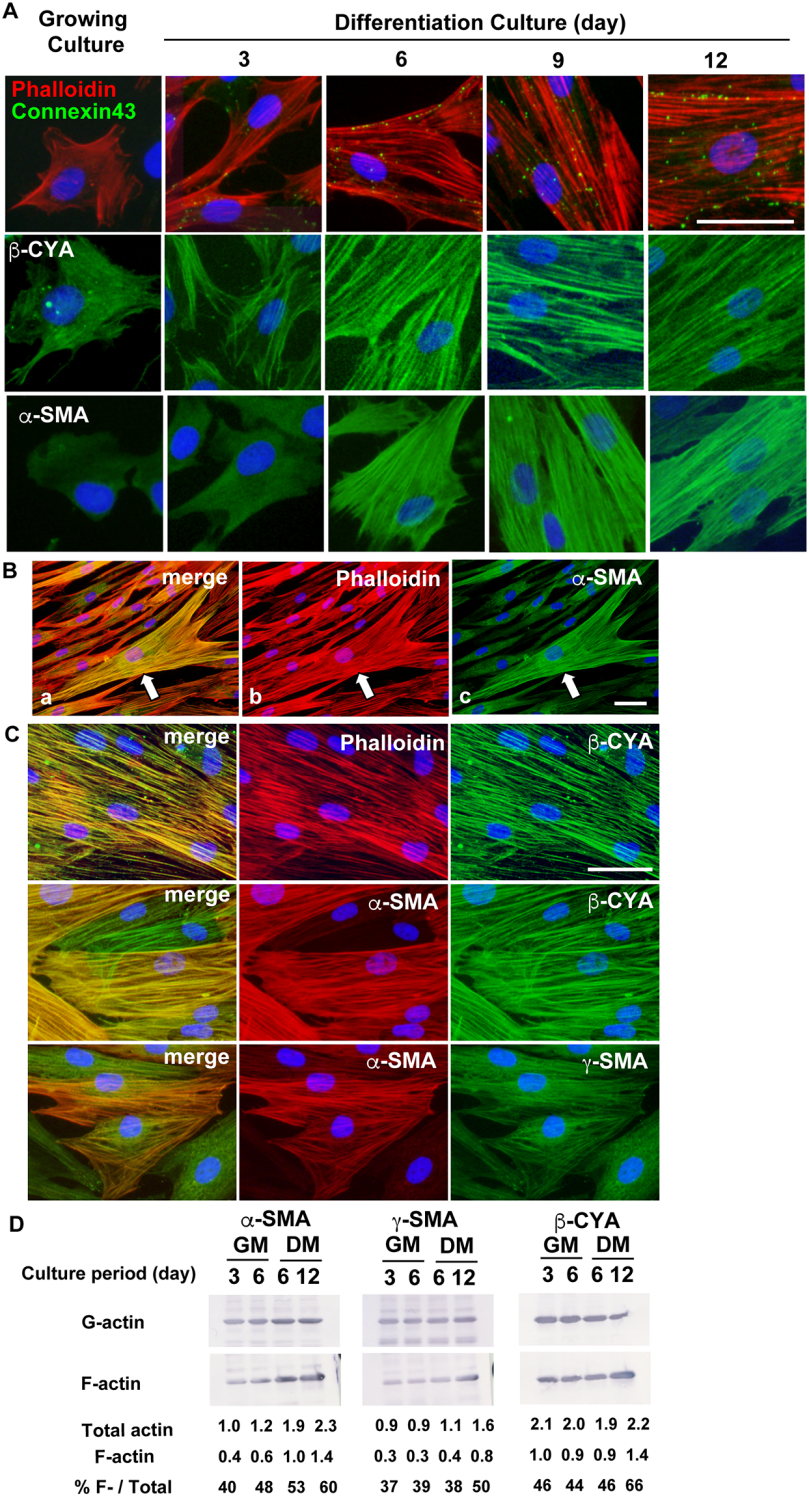
Isoform-dependent reorganization of actin bundles during bladder smooth muscle cell differentiation. (A) hBS11 cells were cultured in pmGM for 3 days or pmDM for 3, 6, 9, and 12 days, and then subjected to immunofluorescence analysis with antibodies for connexin 43 (green in top row), β-CYA (green in middle row) and α-SMA (green in lower row). Filamentous actin was visualized with Alexa 546-conjugated phalloidin (red in upper row). Nuclei were stained with DAPI (blue). Scale bar, 50 μm. (B) hBS11 cells were cultured for 6 days in pmDM and then subjected to immunofluorescent analysis with antibodies for α-SMA (c). Filamentous actin was visualized with Alexa 546-conjugated phalloidin (b). Nuclei were stained with DAPI (blue). The merged image was shown in (a). Arrows represent a hypertrophic cell containing α-SMA-positive actin bundles. Scale bars, 50 μm. (C) hBS11 cells were cultured in pmDM for 12 days and then subjected to immunofluorescence analysis with antibodies for β-CYA (green in upper and middle rows), and α-SMA (red in middle and lower rows), and γ-SMA (green in lower row). Filamentous actin was visualized with Alexa 546-conjugated phalloidin (red in upper row). Nuclei were stained with DAPI (blue). Scale bar, 50 μm. Merged images are shown in the left column. (D) hBS11 cells were cultured in pmGM for 3 and 6 days or pmDM for 6 and 12 days, and then subjected to quantification of globular (G-actin) and filamentous (F-actin) forms. Fractions of G- and F-actin prepared from 10 μg of total proteins were quantified by immunoblotting analysis with isoform-specific antibodies for α-SMA, γ-SMA and β-CYA. The signal intensity of each band was quantified with Image J software and shown as arbitrary units. Total actin represents the sum of [G-actin] and [F-actin], and [% F-/Total] represents the ratio of F-actin in each isoform.

We examined connexin 43 expression to assess the correlation between actin bundle reorganization and the smooth muscle differentiation phenotype. This protein plays a role in the intercellular conduction between neighboring differentiated bladder SMCs but is unrelated to the organization of actin bundles and the contractile apparatus [30]. Specific antibodies against connexin 43 were detected as spotty signals on the surface of hBS11 cells during differentiation but not proliferation (upper row in Fig 3A). The connexin 43 signal increased as actin bundles developed and aligned with the cell-to-cell contact regions of differentiated hBS11 cells. These results suggest that actin bundles are reorganized in conjunction with the appearance of smooth muscle differentiation phenotypes.

To determine the distinct contributions of actin isoforms to the reorganization of actin bundles during smooth muscle differentiation, the subcellular localization of actin isoforms was determined with actin isoform-specific antibodies. β-CYA was distributed in short actin bundles as well as in the cytoplasm of proliferating hBS11 cells (middle row in Fig 3A). β-CYA contributed to actin bundle reorganization during the early stage of differentiation, and β-CYA-positive bundles remained until the cells reached full differentiation. In contrast to β-CYA, a diffuse α-SMA signal was observed in the cytoplasm of proliferating hBS11 cells, and α-SMA did not contribute to the initial development of actin bundles by day 3 of differentiation (lower row in Fig 3A). α-SMA-positive bundles appeared on day 6 in a minor subset of hypertrophic cells (Fig 3B), and these bundles increased during the later stages of differentiation. The majority of cells contained α-SMA-positive bundles on day 12 of differentiation (Fig 3C).

The actin bundles formed during smooth muscle differentiation always contained β-CYA microfilaments (upper row in Fig 3C), whereas only some β-CYA-positive bundles contained α-SMA microfilaments (middle row in Fig 3C). In addition, each differentiated hBS11 cell comprised either β-CYA-positive and α-SMA-negative bundles or β-CYA and α-SMA double-positive bundles. Therefore, differentiated hBS11 cells were classified into subpopulations containing either β-CYA-positive and α-SMA-negative bundles or β-CYA and α-SMA double-positive bundles. Narrow actin bundles contain approximately 10–30 actin microfilaments and wider mats of up to 300 microfilaments [31]. It is assumed that actin microfilaments containing copolymers of actin isoforms do not exist *in vivo* [32–34]. Our results suggest that α-SMA microfilaments are recruited to pre-existing β-CYA-positive bundles during the later stages of SMC differentiation.

γ-SMA exhibited a distribution pattern similar to α-SMA during smooth muscle differentiation (lower row in Fig 3C); however, the level of protein expression was relatively lower. α-skeletal muscle actin (SKA) and cardiac muscle actin (CAA) were not detected in hBS11 cells. The gene expression of γ-cytoplasmic actin (γ-CYA) was markedly decreased during the smooth muscle differentiation of hBS11 cells (S1 Table). A previous study showed that the distribution of γ-CYA in actin bundles was minimal [23]. Thus, α-SKA, CAA and γ-CYA did not contribute to actin bundle reorganization during the smooth muscle differentiation of hBS11 cells.

We assessed the proportion of polymerized (filamentous) actins in hBS11 cells because of its essential role in the reorganization of actin bundles in SMCs [35]. The total α-SMA protein in differentiated hBS11 cells increased to more than twice the level of growing cells. The ratio of filamentous α-SMA also increased from 40% to 60% of the total α-SMA protein (Fig 3D). Thus, the amount of filamentous α-SMA per cell increased more than three-fold during smooth muscle differentiation. Similar results were obtained for γ-SMA. Although the amount of β-CYA remained constant throughout smooth muscle differentiation, the amount of filamentous β-CYA increased in fully differentiated cells during the late stage of differentiation. These results indicate that the enhancement of α-SMA and γ-SMA production and polymerization promotes their recruitment to β-CYA-positive bundles during smooth muscle differentiation.

### Immortalized bladder smooth muscle cells reinstate the physiological functions of smooth muscle cells

**Fig 4:**
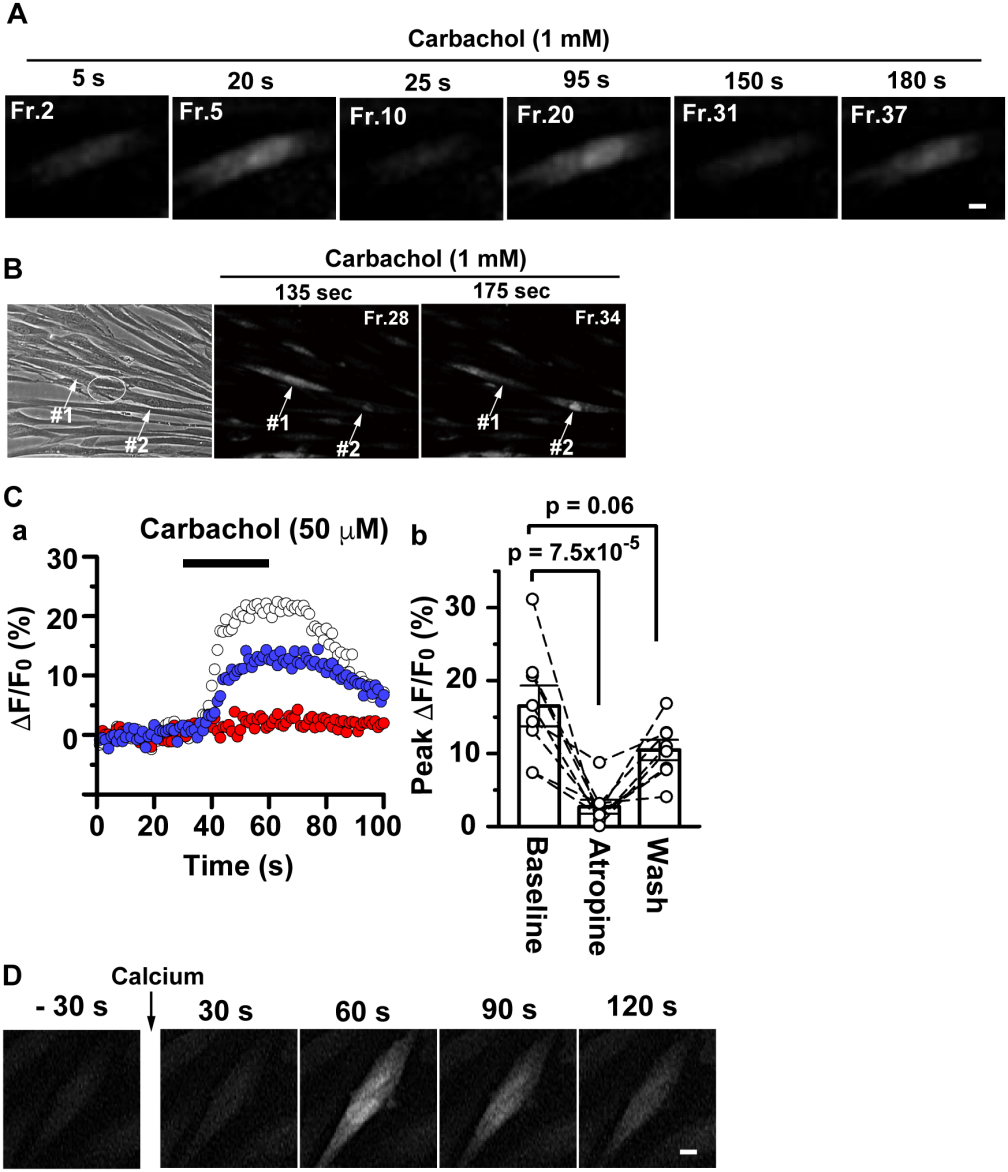
Calcium increase in immortalized human bladder smooth muscle cells. (A) Differentiated hBS11 cells were preloaded with a calcium sensitive dye Fluo-4 AM, and then stimulated with a cholinergic agonist carbachol (1 mM). The cells were observed under epifluorescence microscopy. Fluorescent images correspond to the frames 2, 5, 10, 20, 31, and 37 of S3 Video. Scale bar, 10 μm. (B) Differentiated hBS11 cells were treated as described in (A). The cells were observed under phase contrast and epifluorescence microscopy. The phase contrast image was taken before stimulation with carbachol. Fluorescent images correspond to the frames 28 and 34 of S4 Video. A circle represents a cell-to-cell contact region between neighboring cells named #1 and #2 in a phase contrast image before carbachol stimulation (left panel). Calcium signaling was conducted between neighboring cells (middle and right panels). (C) hBS11 cells were preloaded with Fluo-4 AM for 1 h on day 14 or 19 of differentiation culture and then stimulated with a cholinergic agonist carbachol (50 μM) for 30 s. Digital fluorescent imaging was obtained using a two-photon confocal microscope. ΔF/F_0_ represents percent changes in the fluorescence intensity over resting levels. (a) Effect of the muscarinic receptor antagonist atropine (5 μM) on carbachol-induced intracellular Ca^2+^ elevation. Horizontal bar represents the period of exposure to carbachol. F/F_0_ of hBS11 cells before (open circles), during the treatment of atropine (red circles), and after washing off atropine (blue circles) are shown. (b) Pooled data regarding the effect of atropine on carbachol-induced intracellular Ca^2+^ elevation. Atropine treatment significantly blocked the Ca^2+^ elevation. Statistical significance (p-value) was estimated using the multi-comparison Dunnett’s test (n = 8). Each dashed line connecting open circles represents data obtained from the same cells. Each bar represents average and standard error of mean. (D) hBS11 cells were cultured for 14 days in pmDM and preloaded with Fluo-4 AM. Next, the medium was switched to a calcium-deleted Krebs-Ringer solution supplemented with 90 mM KCl. The cells were sequentially observed using epifluorescence microscopy and time-lapse recordings with a 30-second interval. Incubation time before and after stimulation with calcium (2.8 mM) is shown at the upper panel corners. Scale bar, 10 μm.

Physiological agonists induce the influx of extracellular calcium in SMCs [36, 37], which results in SMC contraction. We explored the functional ability of differentiated hBS11 cells by monitoring calcium signaling with time-lapse recordings. Differentiated hBS11 cells were preloaded with a calcium-sensitive dye Fluo-4 AM and then stimulated with the cholinergic receptor agonist carbachol. A number of cells showed a transient increase in Fluo-4 intensity within 30 seconds of carbachol stimulation, followed by a repeated increase in intensity (Fig 4A; S3 Video).

The plasma membranes of neighboring bladder SMCs are in close contact and electrically coupled via gap junctions; thus, action potentials are conducted from one cell to another [17]. Calcium signaling triggered by carbachol ran across the longitudinal axis of a cell and was then conducted to the neighboring cell (Fig 4B, S4 Video). AChR(M3) expression in differentiated hBS11 cells was relatively low compared with other smooth muscle differentiation-related proteins (Fig 2B). An exclusive subset of bladder SMCs is innervated and respond to acetylcholine in vivo [17]. Thus, the minor subset of hBS11 cells that highly express AChR(M3) may exclusively respond to the agonist.

To determine the role of muscarinic acetylcholine receptors in intracellular calcium elevation on stimulation with carbachol, the increase in intracellular calcium was quantified using a two-photon confocal microscope (S3 Fig). Prominent elevation of Fluo-4 intensity induced by carbachol was prevented by the muscarinic receptor antagonist atropine (Fig 4Ca and 4Cb). The mean percentages of peak fluorescence intensity over resting levels (ΔF/F_0_) in hBS11 cells were 16.5% ± 2.8% and 2.7% ± 1.0% before and during the atropine treatment, respectively, and 7.9% ± 2.7% after washing off atropine. Collectively, the results suggest that differentiated hBS11 cells increase intracellular calcium through the activation of AChR(M3) by carbachol.

The majority of hBS11 cells did not directly respond to carbachol; however, they may retain the ability to generate calcium signaling. To explore this idea, the plasma membranes of hBS11 cells were depolarized with high extracellular concentrations of potassium [38]. Many cells showed calcium signaling within 1 minute of extracellular calcium addition (Fig 4D; S5 Video). Therefore, hBS11 cells retain the ability to promote the influx of extracellular calcium depending on the membrane potential.

**Fig 5:**
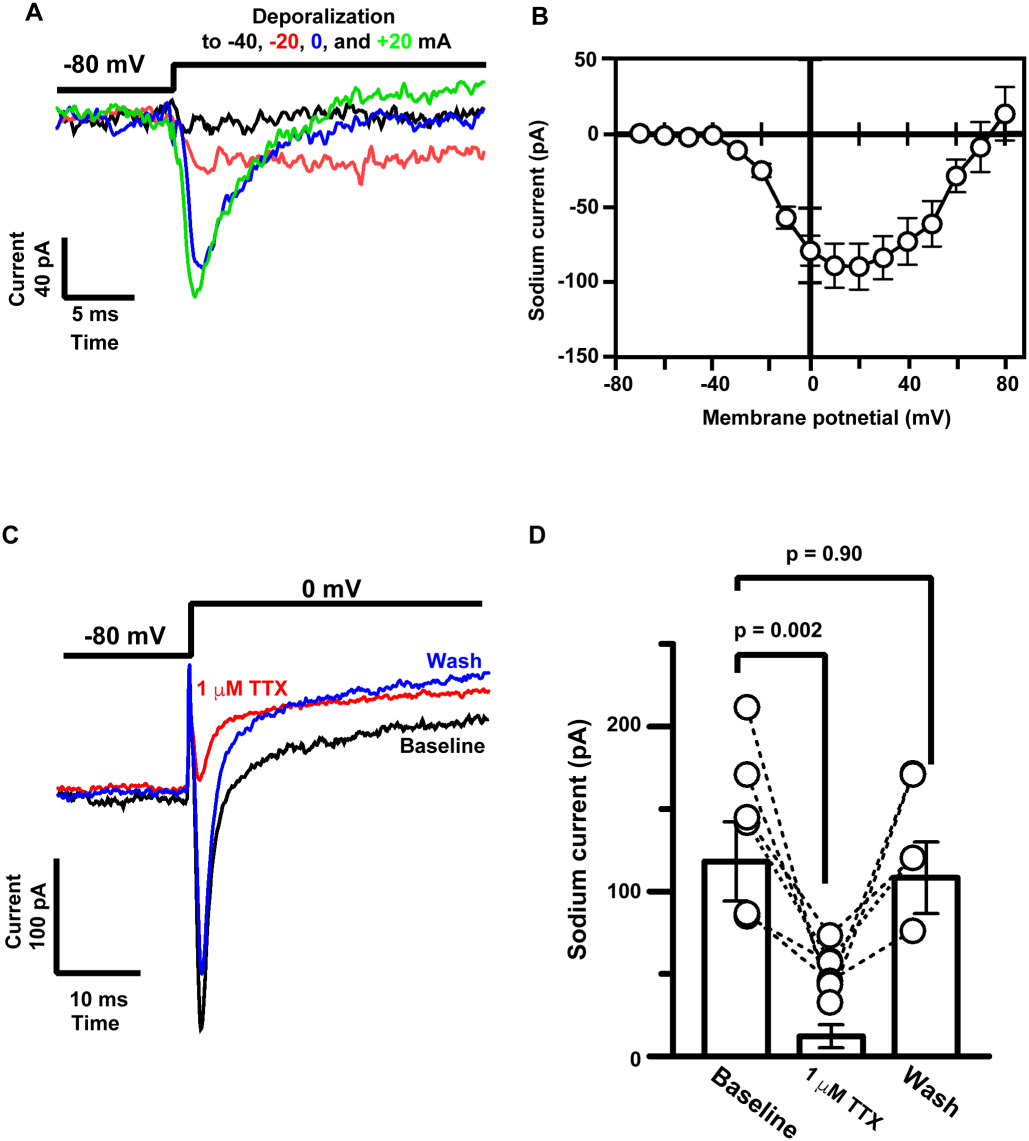
Recordings of voltage-dependent sodium currents during depolarization in immortalized human bladder smooth muscle cells. (A) Voltage-activated sodium currents were recorded by from −80 mV to −40, −20, 0, and 20 mV. (B) Voltage–current relationship of voltage-activated sodium currents in differentiated hBS11 cells (n = 10). Each point represents mean ± standard error of mean. (C) Effect of tetrodotoxin (TTX) on sodium currents. Representative traces of inward currents were elicited by depolarization to 0 mV in hBS11 cells before (black line) and during (red line) TTX treatment and after washing off TTX (blue line). Washing off TTX after 5 min partially recovered the amplitude of inward currents. (D) Summary data for the inhibitory action of TTX on sodium currents. Each dashed line connecting open circles represents data obtained from the same cells. Each bar represents average and standard error of mean.

The membrane excitation of differentiated hBS11 cells was determined because a connection between the electrical action potential and mechanical SMC contraction is essential for controlling the contractility of SMCs. Marked sodium currents were triggered by depolarization from −80 mV to 0 and 20 mV (Fig 5A and 5B). The depolarization-triggered sodium currents were perfectly blocked by the sodium channel blocker tetrodotoxin (Fig 5C and 5D). The results indicate that hBS11 cells reinstated electrophysiological features of SMCs.

### Immortalized bladder smooth muscle cells exhibit contractility depending on calcium signaling

For assessing the ability of differentiated hBS11 cells to contract, the cells were subjected to the collagen gel contraction assay. The gels markedly shrank even in differentiated hBS11 cells that were not stimulated with carbachol probably because the developed actin bundles generated an intense static force in the differentiated cells. Therefore, phasic force generation triggered by carbachol was not detected by the collagen gel contraction assay (data not shown).

Then, to reveal the dynamics of actin bundles in live cells, hBS11 cells were preloaded with the fluorogenic F-actin labeling probe SiR-actin (SiR-A), which comprises jasplakinolide conjugated with silicon rhodamine. Next, the cells were treated with either carbachol or high concentrations of potassium. The time-lapse recordings showed no movement of actin bundles in differentiated hBS11 cells. The movements of actin bundle may be antagonized by the attachment strength of hBS11 cells to the substratum of a dish because the ends of actin bundles are anchored to the plasma membrane. Therefore, the results suggest that the phasic power induced by carbachol is not intense enough to overcome the attachment power of hBS11 cells to the substratum of a dish. This lack of effect was probably the result of insufficient calcium signaling for the force generation required for cell contraction (data not shown).

**Fig 6:**
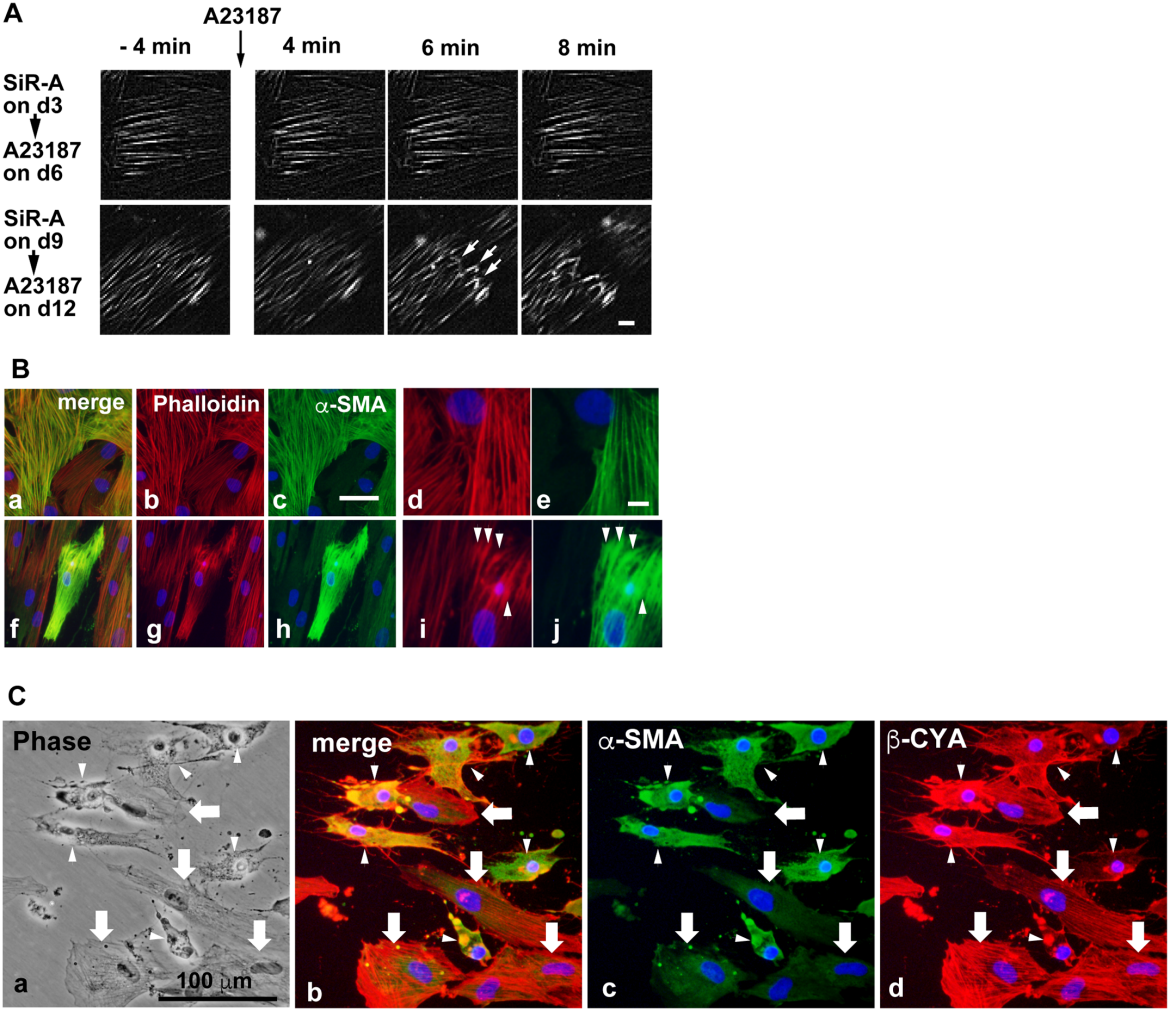
A23187-induced contraction of immortalized human bladder smooth muscle cells. (A) hBS11 cells were preloaded with SiR-actin (100 nM) for 2 h on day 3 or 9 of differentiation culture and then cultured for another 3 days in pmDM. Next, the cells were treated with a calcium ionophore A23187 (5 μM) on day 6 (upper row) or 12 (lower row) of differentiated culture. The cells were sequentially observed using epifluorescence microscopy and time-lapse recordings at 5 s intervals. Small arrows represent contracted actin bundles. Incubation time before and after stimulation with A23187 is shown at the upper panel corners. Scale bar, 10 μm. (B) Cells were cultured in pmDM for 12 days and stimulated with (f-j) or without (a-e) A23187 (5 μM) for 10 min. Next, the cells were subjected to filamentous actin staining with Alexa 546-conjugated phalloidin (red in b, d, g and i) and immunostaining analysis with antibodies for α-SMA (green). Images of the same fields are shown in a-c, d-e, f-h and i-j, respectively. Arrow heads represent thickened knob-like actin bundles. Nuclei were stained with DAPI (blue). Scale bars, 100 μm for (a-c and f-h) and 20 μm for (d,e,i and j). (C) hBS11 cells were cultured in pmDM for 7 days and stimulated with A23187 (5 μM) for 45 min. Next, the cells were subjected to immunostaining analysis with antibodies for α-SMA (green) and β-CYA (red). The phase contrast image (a) and merged image (b) of c and d are shown. The cells that were intensely stained with α-SMA antibody (arrow heads) exclusively shrank. In contrast, the intact spreading cells (arrows) contained β-CYA-positive and α-SMA-negative bundles. Scale bar, 100 μm.

The calcium ionophore A23187 markedly increases intracellular calcium concentrations and induces smooth muscle contraction [39, 40]. Therefore, hBS11 cells were preloaded with SiR-A, and then treated with A23187 on day 6 or 12 of differentiation-inducing culture conditions. A23187 showed no effect on the movement of SiR-A-labeled actin bundles in the majority of differentiating cells on day 6 (upper row in Fig 6A; S6 Video). In contrast, A23187 triggered the contraction of actin bundles and the shrinkage of day 12 differentiated cells within 10 minutes of application (lower row in Fig 6A; S7 Video). α-SMA-positive bundles were found in a minor subset of cells on days 6–7 and in most cells on day 12. Thus, the recruitment of α-SMA to actin bundles is involved in the contraction of hBS11 cells.

To determine if α-SMA-positive bundles play a crucial role in cell contraction, differentiated hBS11 cells cultured under differentiation-inducing conditions were stimulated with A23187 for 10 minutes and then probed with α-SMA-specific antibodies. In untreated controls, α-SMA-positive and -negative bundles showed a similar length and thickness (Fig 6Ba–6Be). Cells containing α-SMA-positive bundles contracted when stimulated with A23187 (Fig 6Bf–6Bj). Furthermore, α-SMA-positive bundles showed thickened knob-like ends (Fig 6Bi and 6Bj), which may correspond to the ends of contracted actin bundles (Fig 6A). In addition, an exclusive subset of cells that robustly expressed α-SMA shrunk on differentiation day 7 after treatment with A23187 (Fig 6C). In contrast, other cells containing α-SMA-negative bundles were unaffected. These results indicate that α-SMA-positive bundles play a critical role in contractile properties of bladder smooth SMCs.

### Differentiated bladder smooth muscle cells undergo retrograde differentiation and a restoration of proliferation ability

**Fig 7:**
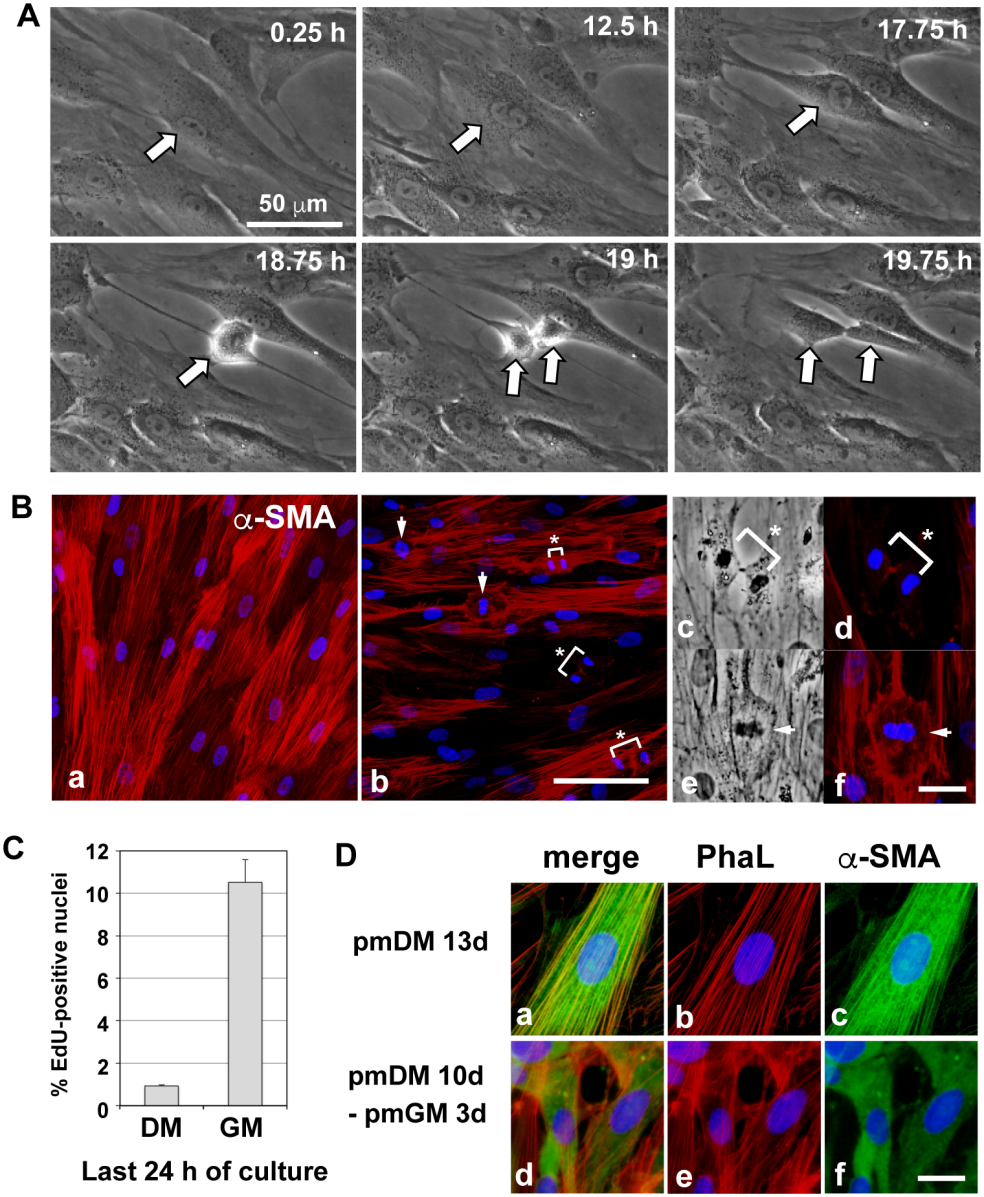
Retrograde differentiation of differentiated immortalized human bladder smooth muscle cells. (A) hBS11 cells were cultured in pmDM for 10 days and then re-fed with pmGM. The cells were sequentially observed using phase contrast microscopy and time-lapse recordings with a 15-min interval. The time after medium switching to pmGM is shown in each image. Arrows represent a differentiated cell and its daughter cells. (B) hBS11 cells were cultured in pmDM for 19 days, and the medium was switched to fresh pmDM (a) or pmGM (b-f). Next, the cells were cultured for another 28 h and subjected to immunostaining analysis with an antibody for α-SMA (red). Panels with high magnification (c-f) show anaphase (c and d) and metaphase (e and f) cells in the re-feeding culture using epifluorescence (d and f) and phase contrast (c and e) microscopy. Asterisks represent telophase chromosomes. Arrows represent metaphase chromosomes. Nuclei were stained with DAPI (blue). Scale bar, 100 μm (a and b), 25 μm (c-f). (C) hBS11 cells were cultured in pmDM for 12 days. The medium was switched to pmDM (DM) or pmGM (GM) and cultured for another 24 h. The cells were labeled with EdU during the last 4 h of culture. EdU-positive nuclei were detected in three independent cultures. The average and standard deviation are analyzed using Student’s *t*-test. The p-value was less than 0.01. (D) Cells were cultured in pmDM for 10 days, and the medium was switched to pmDM (a-c) or pmGM (d-f). Next, the cells were cultured for 3 days and subjected to filamentous actin staining with Alexa 546-conjugated phalloidin (PhaL; red) and immunostaining analysis with antibodies for α-SMA (green). Nuclei were stained with DAPI (blue).

Differentiated contractile SMCs are considered to be non-proliferating; however, these cells retain their proliferative ability [1, 2]. We explored the retrograde differentiation of hBS11 cells from a differentiated state to a proliferating, undifferentiated state. Differentiated hBS11 cells exhibited hypertrophy and a non-proliferative phenotype (Fig 7A). Time-lapse recordings showed that non-proliferating, hypertrophied cells became smaller and resumed mitosis within 24 h after switching the culture medium from pmDM to pmGM (Fig 7A; S8 Video). The extensively developed actin bundles in differentiated cells decreased, and many mitotic cells appeared after re-feeding with growth medium (Fig 7B). DNA synthesizing cells were significantly increased 24 h after re-feeding (Fig 7C). Differentiated cells containing α-SMA-positive bundles were converted to undifferentiated cells, and α-SMA diffused within the cytoplasm during the first three days of re-feeding (Fig 7D). α-SMA, *h*-caldesmon, and calponin were decreased during retrograde differentiation and then increased during re-differentiation (S4 Fig). Thus, differentiated hBS11 cells maintain their plasticity to undergo retrograde differentiation.

### Bladder smooth muscle cells show a gene expression profile distinct from contractile and synthetic vascular smooth muscle cells

Vascular SMCs perform both contractile and synthetic functions [1–3]. Contractile vascular SMCs are spindle-shaped cells that can be converted to a synthetic phenotype featuring the constitutive secretion of collagen type I and large amounts of synthetic organelles. Synthetic vascular SMCs exhibit a rhomboid morphology and higher proliferating rate than contractile cells; however, these cells irreversibly lose contractile structures and abilities during *in vitro* culturing. The synthetic cells that lose their differentiation potential are called dedifferentiated cells. Undifferentiated hBS11 cells retained the potential to differentiate into contractile SMCs. We noted both morphological and functional similarities between undifferentiated hBS11 cells and synthetic vascular SMCs.

To determine if the reversible differentiation of hBS11 cells is similar to the modulation between synthetic and contractile phenotypes, the gene expression profiles of undifferentiated and differentiated hBS11 cells were determined using DNA array analyses (S1 Table). A gene ontology analysis revealed that the expression of extracellular matrix-related genes was greatly induced in differentiated hBS11 cells (Table 1; S2 and S3 Tables). For example, tenascin XB, collagen XIV (α1), and matrix Gla proteins were markedly upregulated (S4 Table). In contrast, cell cycle-related genes and organelle dynamics-related genes were downregulated in differentiated hBS11 cells (Table 2; S2 and S4 Tables). The DNA array analysis showed that collagen type I (α1 and α2) genes were highly expressed in both undifferentiated and differentiated hBS11 cells; however, the expression levels increased approximately two-fold during differentiation (S3 Table). Differentiated hBS11 cells robustly expressed collagens and other extracellular matrix materials. These results indicate that the conversion between differentiated and undifferentiated hBS11 cells is distinct from the phenotype switch from differentiated/contractile to dedifferentiated/synthetic vascular SMCs.

**Table 1:** Gene ontology analysis of upregulated genes in differentiated hBS11 cells.

GO ACCESSION	GO Term
GO:0031012	extracellular matrix
GO:0005578	proteinaceous extracellular matrix
GO:0044421	extracellular region part
GO:0043062	extracellular structure organization
GO:0030198	extracellular matrix organization
GO:0005576	extracellular region
GO:0005615	extracellular space
GO:0012505	endomembrane system
GO:0044420	extracellular matrix component
GO:0031982	vesicle

**Table 2:** Gene ontology analysis of downregulated genes in differentiated hBS11 cells.

GO ACCESSION	GO Term
GO:0022402	cell cycle process
GO:0043233	organelle lumen
GO:0043232	intracellular non-membrane-bounded organelle
GO:0007049	cell cycle
GO:0048285	organelle fission
GO:0007067	mitotic nuclear division
GO:0000280	nuclear division
GO:0005694	chromosome
GO:0043228	non-membrane-bounded organelle
GO:0006996	organelle organization

### Retrograde differentiation of human bladder smooth muscle cells provides a novel mechanistic view of bladder smooth muscle function and regeneration

**Fig 8:**
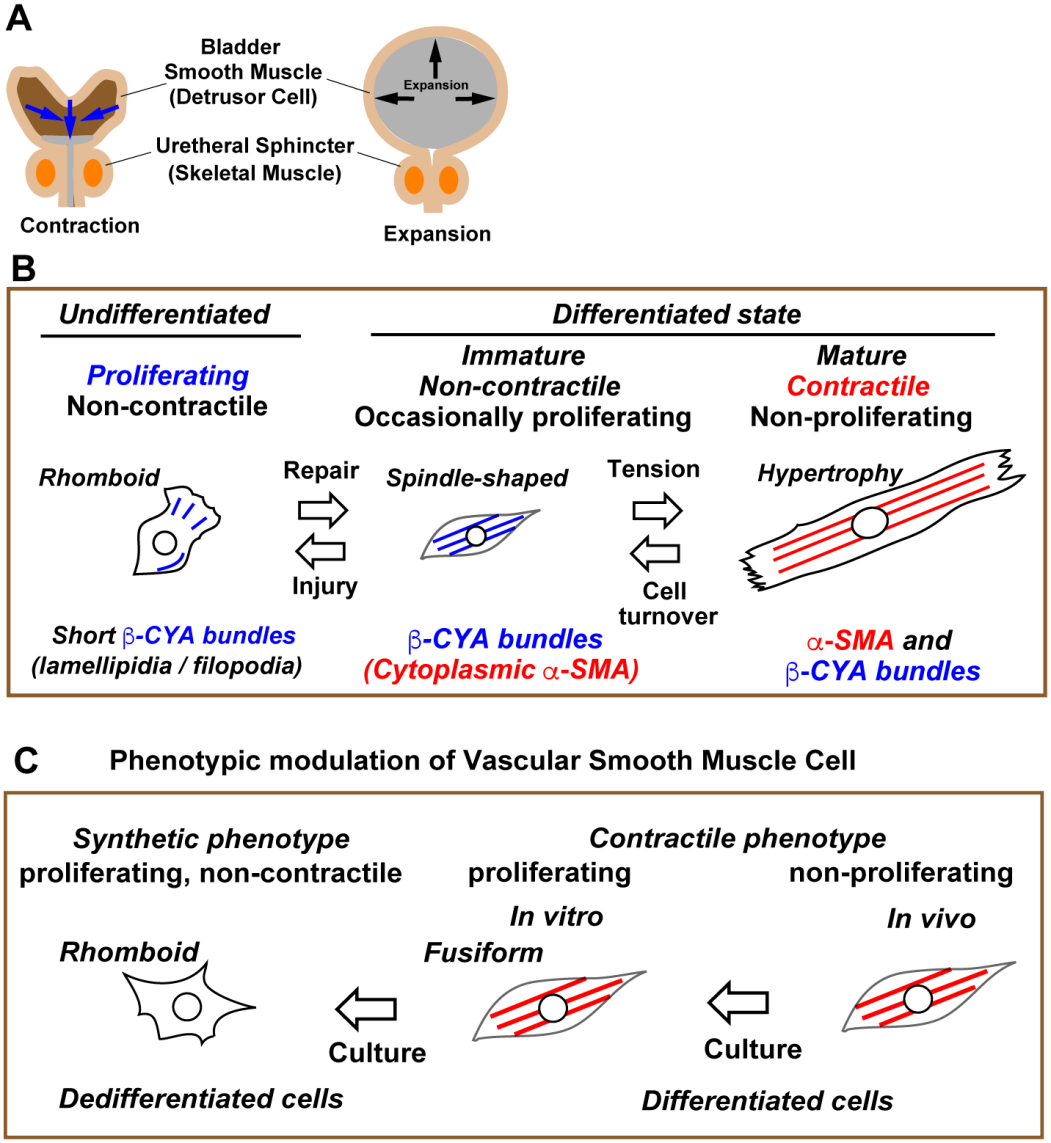
Phenotypic modulation of human bladder smooth muscle cells. (A) Structural changes of a bladder between contraction for expelling and expansion for storage of urine. Arrows represent the direction of intraluminal pressure. (B) A schematic figure for bidirectional phenotypic modulation of human bladder smooth muscle cells. Detailed explanations and discussion for reversible differentiation and isoform-dependent reorganization of actin bundles are outlined in the main text. (C) A schematic figure for the phenotypic modulation of vascular smooth muscle cells. Dedifferentiated SMC is a collective term of a variety of SMC subtypes. Dedifferentiation from contractile phenotype to synthetic phenotype is often irreversible or partially reversible.

The present study indicates three distinct states of human bladder SMCs: undifferentiated/proliferating, immaturely differentiated/non-contractile, and fully differentiated/contractile (Fig 8A and 8B). hBS11 cells showed the bidirectional oscillation of phenotypes, which is completely dependent on culture conditions. In contrast to previous studies, a dedifferentiated phenotype (Fig 8C) was not observed in hBS11 cells. Analysis of the differentiation procedure of hBS11 cells suggests that α-SMA-positive bundles play a crucial role in the reinstatement of contractile properties in bladder SMCs.

## Discussion

Immortalization of HBdSMCs by expressed CDK4, cyclin D1, and telomerase suggests that growth arrest of primary cultured SMCs is due to the activation of Rb because CDK4 and cyclinD1 give rise to a protein complex that inactivates Rb by its protein kinase activity (S5 Fig).

We have provided direct evidence of the switch between differentiated and undifferentiated states in bladder SMCs. The newly established hBS11cell line exhibited a stable phenotype of undifferentiated SMCs that retain their differentiation potential. This SMC system enables the study of smooth muscle differentiation mechanisms *in vitro*. In addition, the optimized growth medium pmGM caused fully differentiated SMCs to undergo retrograde differentiation and resume mitosis. hBS11 cells are a unique SMC system that reproduces the bidirectional phenotypic modulation of SMCs.

Our results suggest that the dynamic remodeling of actin bundles plays an essential role in the differentiation of bladder SMCs. Isoform-specific antibodies revealed the sequence of events during actin bundle reorganization. First, β-CYA microfilaments formed actin bundles under smooth muscle differentiation-inducing conditions. Next, α-SMA microfilaments were recruited to pre-existing actin bundles. The α-SMA-dependent dynamics of actin bundles in hBS11 cells indicates that the recruitment of α-SMA microfilaments to actin bundles is a critical event for establishing contractility in bladder SMCs. Despite their highly conserved amino acid sequence, actin isoforms cannot substitute for each other and are functionally specialized [33]. α-SMA but not β-CYA mediates myofibroblast contraction, even though both actin isoforms colocalize in bundles of actin stress fibers [41]. α-SMA microfilaments may play an essential role in the contractile apparatus of differentiated hBS11 cells. In addition, the recruitment of α-SMA depends on the cellular context because each hBS11 cell contained either α-SMA-positive or –negative actin bundles but not both. Therefore, the re-localization of α-SMA in actin bundles may be a programmed event during smooth muscle differentiation.

We hypothesize that pre-existing β-CYA bundles aid in determining the alignment of the contractile apparatus. β-CYA bundles generated during the early period of differentiation are susceptible to mechanical strain and can be rearranged to antagonize mechanical stress. In differentiating bladder SMCs, β-CYA bundles may determine the direction of force generation by the SMCs because α-SMA microfilaments were recruited, probably to generate the contractile apparatus within pre-existing actin bundles. How the contractile apparatus is organized in SMCs remains unknown. However, the reorganization of the contractile apparatus in hBS11 cells will soon be elucidated using an improved unroofing method with atomic force microscopy [42].

We identified three distinct states of hBS11 cells: undifferentiated and proliferating, immaturely differentiated, and fully differentiated and contractile. Undifferentiated cells divided very rapidly and extensively, whereas immaturely differentiated cells at a transient stage of differentiation exhibited limited proliferation capacity. Thus, undifferentiated cells play a role in production of a large pool of SMCs upon severe smooth muscle tissue injury, resulting in the regeneration of bladder smooth muscles. The immaturely differentiated cells may be important for cell replacement or turnover to maintain tissue homeostasis by producing small numbers of new cells. It will be interesting to determine if the present scheme for bladder SMCs is applicable to the phenotypic modulation of vascular SMCs.

In contrast to differentiated vascular SMCs, fully differentiated bladder SMCs underwent hypertrophy and synthesized extracellular matrix materials. The large changes in bladder volume during the storage of urine are coupled with changes in bladder SMCs size [17]. Bladder hypertrophy is associated with an increase in extracellular materials, including collagen [17]. Therefore, the hypertrophy and synthetic nature of fully differentiated hBS11 cells *in vitro* likely corresponds to the characteristics of bladder SMCs *in vivo*. Mechanical microenvironment may be involved in the phenotypic modulation of bladder SMCs *in vivo*. From this point of view, we note that the smooth muscle differentiation of hBS11 cells is analogous to the differentiation from proto-myofibroblasts to myofibroblasts [43].

Collectively, the virtually immortalized human bladder SMC line hBS11 retains the ability of retrograde differentiation. hBS11 cells are a breakthrough tool for the study of the bidirectional phenotypic modulation accompanied by the generation and degradation of the contractile apparatus in SMCs. Notably, hBS11 cells can help elucidate the regulatory mechanisms of isoform-specific actin functions. Therefore, this unique cell line opens new avenues for mechanistic research on the phenotypic modulation of human SMCs and therapeutic interventions for human bladder diseases.

## Supporting information

S1 Fig Primary cultured human bladder smooth muscle cells undergo hypertrophy. A postmitotic compact cell in the parental HBdSMC culture at passage 6 underwent hypertrophy and then gave rise to an extensively spreading cell. The cell was sequentially observed using phase-contrast microscopy and time-lapse recordings with a 15-min interval. Lines represent the positions of both ends of the cell. Scale bar, 100 μm.
(TIF)

S2 Fig Hypertrophic hBS11 cells exhibit a differentiation phenotype even in postconfluent culture. (A) hBS11 cells were grown to confluency for 10 days in pmGM. The cells were subjected to immunofluorescence analysis with antibodies for α-SMA (green). Filamentous actin was stained with Alexa 546-conjugated phalloidin (red). Nuclei were counterstained with DAPI (blue). A hypertrophic cell was superimposed on a basal cell layer and exhibited α-SMA-positive bundles. (B) Speculation on the hypertrophy of superimposed cells in postconfluent culture. (Upper panel) A low cell density enables cells to undergo hypertrophy. (Lower panel) Occasional cell division in postconfluent culture may exclude one of the daughter cells from the basal cell layer. The superimposed cell (arrow) spreads over the basal cell layer and underwent hypertrophy (red).
(TIF)

S3 Fig Carbachol-induced increase of intracellular calcium in human bladder smooth muscle cells. hBS11 cells were cultured and treated as described in Fig 4C. (A–C) Differentiated hBS11 cells (A) were preloaded with Fluo-4, and digital fluorescent images were obtained before (B) and during stimulation with carbachol (C). Arrowheads represent the region of interest within the cells. The carbachol-containing solution was flushed through a glass pipette [shown on left side of the field in (A)]. (D) Percentage of fluorescence intensity over resting level (ΔF/F_0_) in hBS11 cells after stimulation with carbachol for 30 s. Each symbol represents the average and standard error of the mean.
(TIF)

S4 Fig Retrograde differentiation and re-differentiation of immortalized human bladder smooth muscle cells. (A) Schematic figure of retrograde differentiation and re-differentiation of hBS11 cells. hBS11 cells were cultured in pmDM for 9 d, and then medium was switched to pmGM and further cultured for 3more d. The cells were replated in pmGM, then cultured for 12 days in pmDM. (B) hBS11 cells were cultured in pmGM for 3 days (pmGM 3d) or pmDM for 9 days (pmDM 9d). Then the medium was switched to pmGM again for retrograde differentiation, and further cultured for 24 h (pmDM + pmGM 24 h), 48 h (pmDM + pmGM 48 h). The cells were replated on day 3 of retrograde differentiation culture, then cultured in pmGM for 3 d (Replate + pmGM 3d) or pmDM for 12 d (Replate + pmDM 12d). Ten or 20 (for calponin) micrograms of total protein was subjected to immunoblotting analysis with antibodies for α-smooth muscle actin (α-SMA), myosin heavy chain 11 (MYH11), h-caldesmon, calponin, and β-tubulin. l-Calponin is an isoform of calponin (calponin 1) and whose expression is primarily restricted to urogenital tissues (Draeger et al., FEBS Lett. 291, 24–28, 1991).
(TIF)

S5 Fig Accumulation of hypophosphorylated retinoblastoma protein during differentiation of immortalized human bladder smooth muscle cells. hBS11 cells were cultured in pmGM (GM) for 3 and 6 days or pmDM (DM) for 6, 10, and 12 days. The cells have reached confluence on day 6 of culture in pmGM. Ten micrograms of total protein was subjected to immunoblotting analysis with antibodies for retinoblastoma protein (Rb) and β-tubulin. U, upper band containing hyperphosphorylated Rb protein; L, lower band containing hypophosphorylated Rb protein.
(TIF)

S1 Table Results of DNA array analysis (75 percentile). (XLS)

S2 Table Results of gene ontology analysis. (XLS)

S3 Table Genes up-regulated during smooth muscle differentiation. Genes whose expression levels were increased by more than 100% in differentiated hBS11 cells are shown.
(XLS)

S4 Table Genes down-regulated during smooth muscle differentiation. Genes whose expression levels were decreased by more than 50% in differentiated hBS11 cells are shown.
(XLS)

S1 Video Heterogeneous subpopulations in primary cultured human bladder smooth muscle cells (HBdSMCs). The parental HBdSMC culture contained heterogeneous subpopulations at passage 6: proliferating/compact cells and non-proliferating/extensively spreading cells. The cells were sequentially observed using phase-contrast microscopy and time-lapse recordings with a 15-min interval. The sequence duration was 72 h.
(AVI)

S2 Video Rapid division of immortalized human bladder smooth muscle cells. The cells were sequentially observed using phase-contrast microscopy and time-lapse recordings with a 15-min interval. The sequence duration was 68 h.
(AVI)

S3 Video Carbachol-induced repeated calcium uptake in immortalized human bladder smooth muscle cells. hBS11 cells were cultured for 12 days in pmDM. Differentiated hBS11 cells were preloaded with the calcium-sensitive dye Fluo-4 AM and then stimulated with the cholinergic agonist carbachol (1 mM). The cells were sequentially observed using epifluorescence microscopy and time-lapse recordings with a 5-s interval. The sequence duration was 5 min and began with the addition of carbachol.
(AVI)

S4 Video Intercellular transmission of carbachol-triggered calcium wave. **h**BS11 cells were treated as described in S3 Video. The cells were sequentially observed using epifluorescence microscopy and time-lapse recordings with a 5-s interval. Calcium signaling moved from left to right along the long axis of several cells. The calcium wave was then transmitted to neighboring cells. The sequence duration was 5 min and began with the addition of carbachol.
(AVI)

S5 Video High potassium-induced calcium uptake. **hBS11 cells were cultured for 14 days in pmDM and preloaded with Fluo-4 AM**. Next, the medium was switched to a calcium-deleted Krebs-Ringer solution supplemented with 90 mM KCl. The cells were sequentially observed using epifluorescence microscopy and time-lapse recordings with a 30-s interval. The sequence duration was 15 min and began with the addition of calcium (2.8 mM).
(AVI)

S6 Video No effect of A23187 on immaturely differentiated hBS11 cells. hBS11 cells were preloaded with SiR-actin for 2 h on day 3 of differentiation-inducing culture. Next, the cells were cultured in pmDM and stimulated with A23187 (5 μM) on day 6. The cells were sequentially observed using epifluorescence microscopy and time-lapse recordings with a 30-s interval. The sequence duration was 10 min and began with the stimulation by A23187.
(AVI)

S7 Video A23187-induced contraction of actin bundles in fully differentiated hBS11 cells. hBS11 cells were preloaded with SiR-actin for 2 h on day 9 of differentiation-inducing culture. Next, the cells were further cultured in pmDM and stimulated with A23187 (5 μM) on day 12. The cells were sequentially observed using epifluorescence microscopy and time-lapse recordings with a 30-s interval. The sequence duration was 10 min and began with the stimulation by A23187. The magnification is the same as Video 6.
(AVI)

S8 Video Mitosis resumed in hBS11 cells after the addition of re-feeding growth medium. hBS11 cells were cultured for 10 days in pmDM. Next, the medium was switched to pmGM. The cells were sequentially observed using phase-contrast microscopy and time-lapse recordings with a 15-min interval. The sequence duration was 48 h and began when the medium was switched to pmGM.
(AVI)
