# Supplementary figures and images for: Reversible differentiation of immortalized human bladder smooth muscle cells accompanied by actin bundle reorganization
Source: PLoS One. 2017 Oct 19;12(10):e0186584. doi: 10.1371/journal.pone.0186584 (PMC5648286; doi:10.1371/journal.pone.0186584)

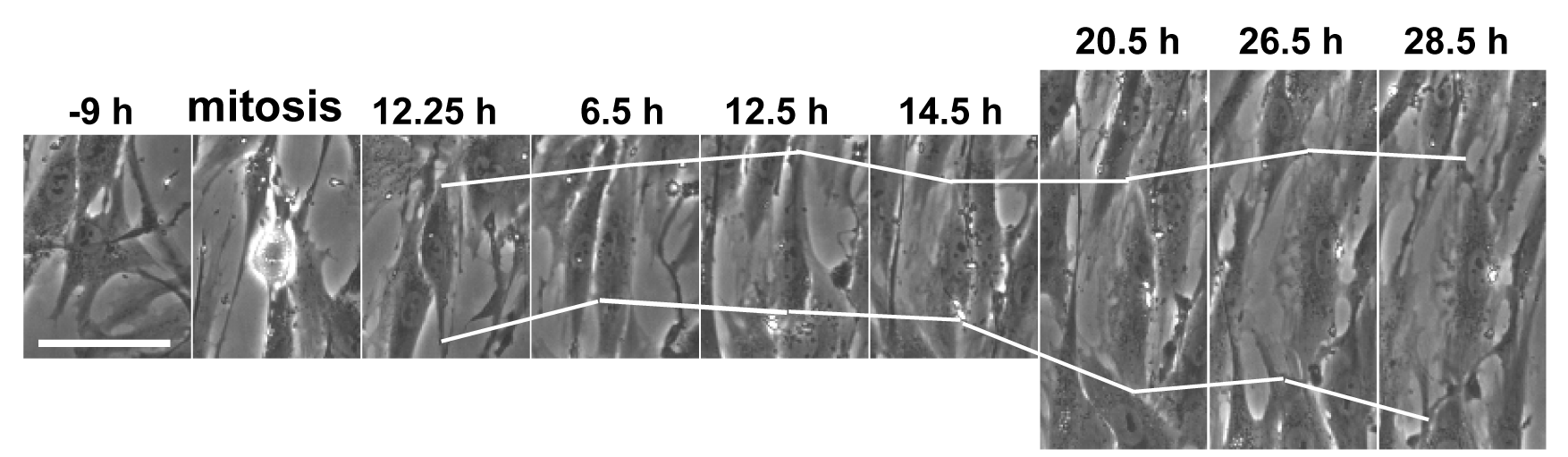

Supplement: S1 Fig — A postmitotic compact cell in the parental HBdSMC culture at passage 6 underwent hypertrophy and then gave rise to an extensively spreading cell. The cell was sequentially observed using phase-contrast microscopy and time-lapse recordings with a 15-min interval. Lines represent the positions of both ends of the cell. Scale bar, 100 μm. (TIF) [file pone.0186584.s001.tif]

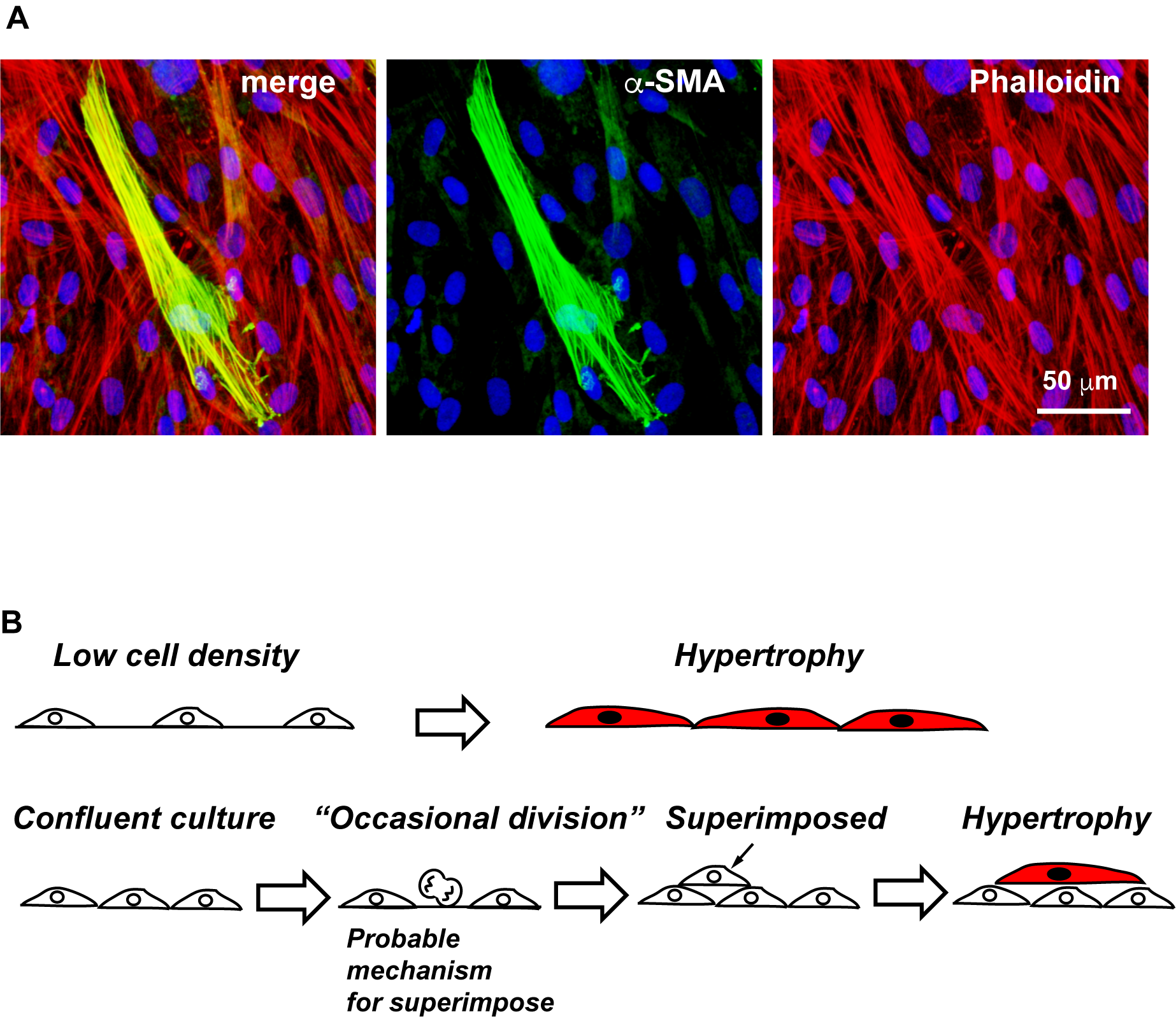

Supplement: S2 Fig — (A) hBS11 cells were grown to confluency for 10 days in pmGM. The cells were subjected to immunofluorescence analysis with antibodies for α-SMA (green). Filamentous actin was stained with Alexa 546-conjugated phalloidin (red). Nuclei were counterstained with DAPI (blue). A hypertrophic cell was superimposed on a basal cell layer and exhibited α-SMA-positive bundles. (B) Speculation on the hypertrophy of superimposed cells in postconfluent culture. (Upper panel) A low cell density enables cells to undergo hypertrophy. (Lower panel) Occasional cell division in postconfluent culture may exclude one of the daughter cells from the basal cell layer. The superimposed cell (arrow) spreads over the basal cell layer and underwent hypertrophy (red). (TIF) [file pone.0186584.s002.tif]

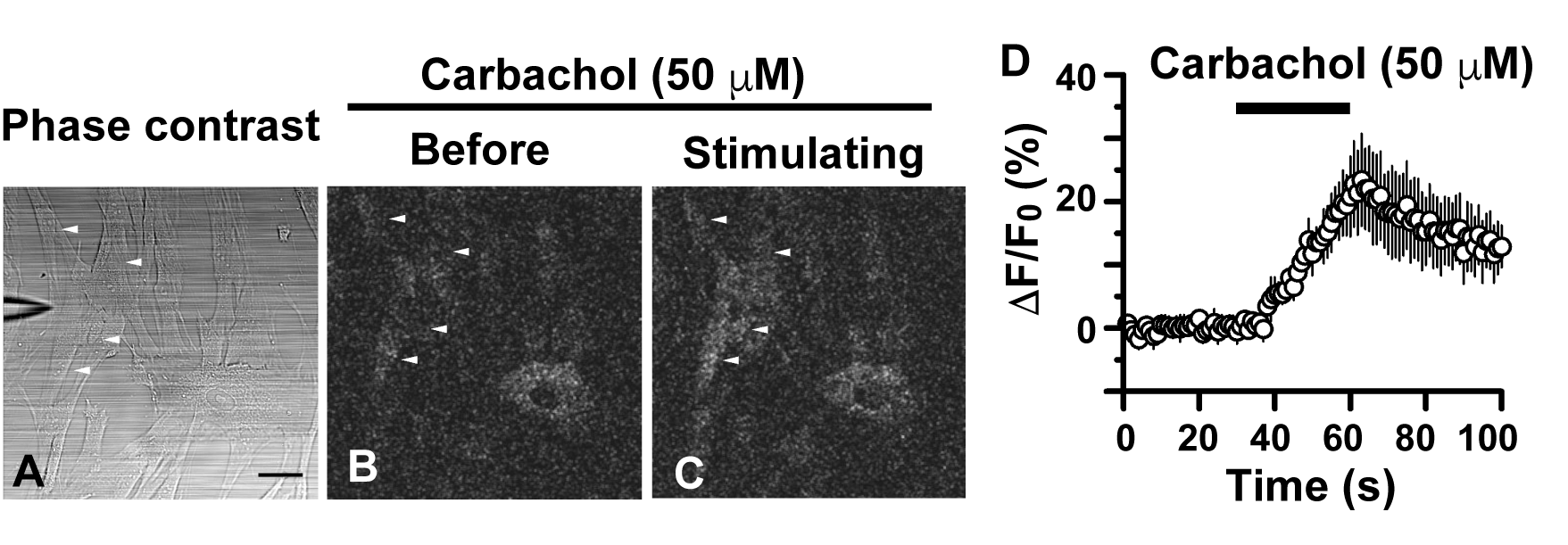

Supplement: S3 Fig — hBS11 cells were cultured and treated as described in Fig 4C. (A–C) Differentiated hBS11 cells (A) were preloaded with Fluo-4, and digital fluorescent images were obtained before (B) and during stimulation with carbachol (C). Arrowheads represent the region of interest within the cells. The carbachol-containing solution was flushed through a glass pipette [shown on left side of the field in (A)]. (D) Percentage of fluorescence intensity over resting level (ΔF/F0) in hBS11 cells after stimulation with carbachol for 30 s. Each symbol represents the average and standard error of the mean. (TIF) [file pone.0186584.s003.tif]

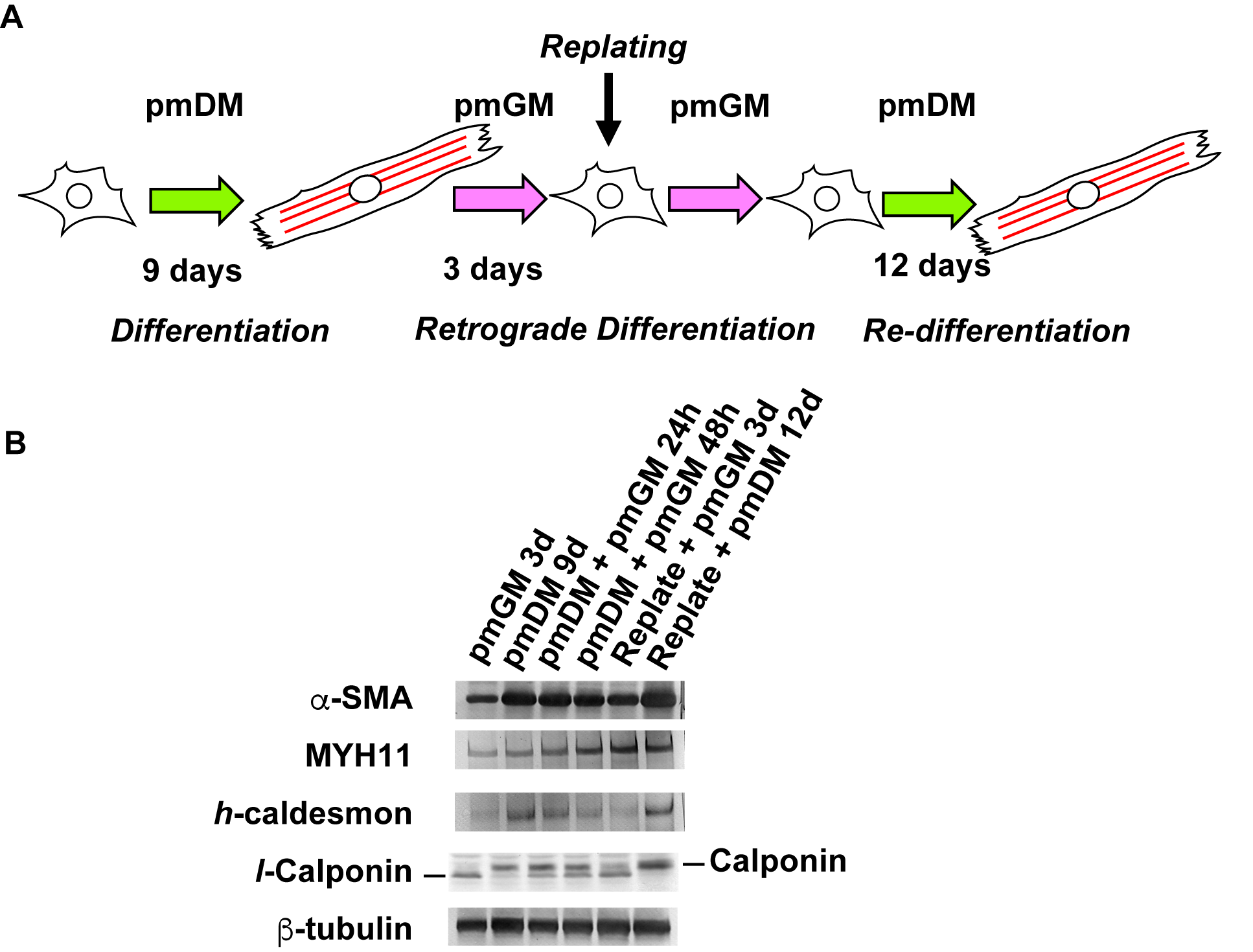

Supplement: S4 Fig — (A) Schematic figure of retrograde differentiation and re-differentiation of hBS11 cells. hBS11 cells were cultured in pmDM for 9 d, and then medium was switched to pmGM and further cultured for 3more d. The cells were replated in pmGM, then cultured for 12 days in pmDM. (B) hBS11 cells were cultured in pmGM for 3 days (pmGM 3d) or pmDM for 9 days (pmDM 9d). Then the medium was switched to pmGM again for retrograde differentiation, and further cultured for 24 h (pmDM + pmGM 24 h), 48 h (pmDM + pmGM 48 h). The cells were replated on day 3 of retrograde differentiation culture, then cultured in pmGM for 3 d (Replate + pmGM 3d) or pmDM for 12 d (Replate + pmDM 12d). Ten or 20 (for calponin) micrograms of total protein was subjected to immunoblotting analysis with antibodies for α-smooth muscle actin (α-SMA), myosin heavy chain 11 (MYH11), h-caldesmon, calponin, and β-tubulin. l-Calponin is an isoform of calponin (calponin 1) and whose expression is primarily restricted to urogenital tissues (Draeger et al., FEBS Lett. 291, 24–28, 1991). (TIF) [file pone.0186584.s004.tif]

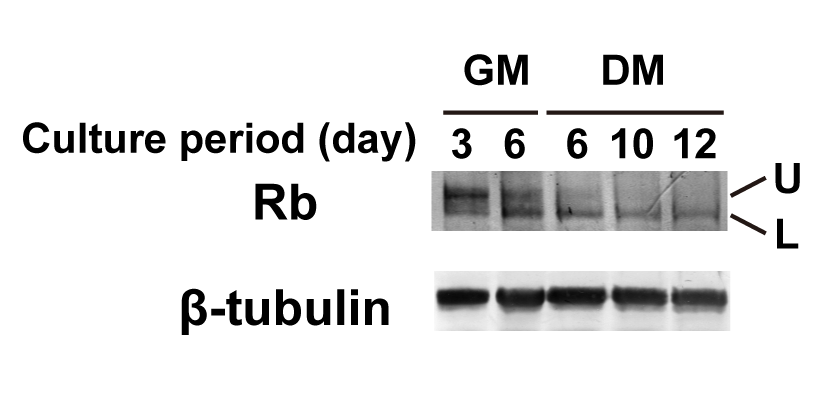

Supplement: S5 Fig — hBS11 cells were cultured in pmGM (GM) for 3 and 6 days or pmDM (DM) for 6, 10, and 12 days. The cells have reached confluence on day 6 of culture in pmGM. Ten micrograms of total protein was subjected to immunoblotting analysis with antibodies for retinoblastoma protein (Rb) and β-tubulin. U, upper band containing hyperphosphorylated Rb protein; L, lower band containing hypophosphorylated Rb protein. (TIF) [file pone.0186584.s005.tif]
